# Supplementary material for: Gut insulin action protects from hepatocarcinogenesis in diabetic mice comorbid with nonalcoholic steatohepatitis
Source: Nat Commun. 2023 Oct 18;14:6584. doi: 10.1038/s41467-023-42334-y (PMC10584811; doi:10.1038/s41467-023-42334-y)
Supplement: Supplementary file 1 — Supplementary Information [file 41467_2023_42334_MOESM1_ESM.pdf]

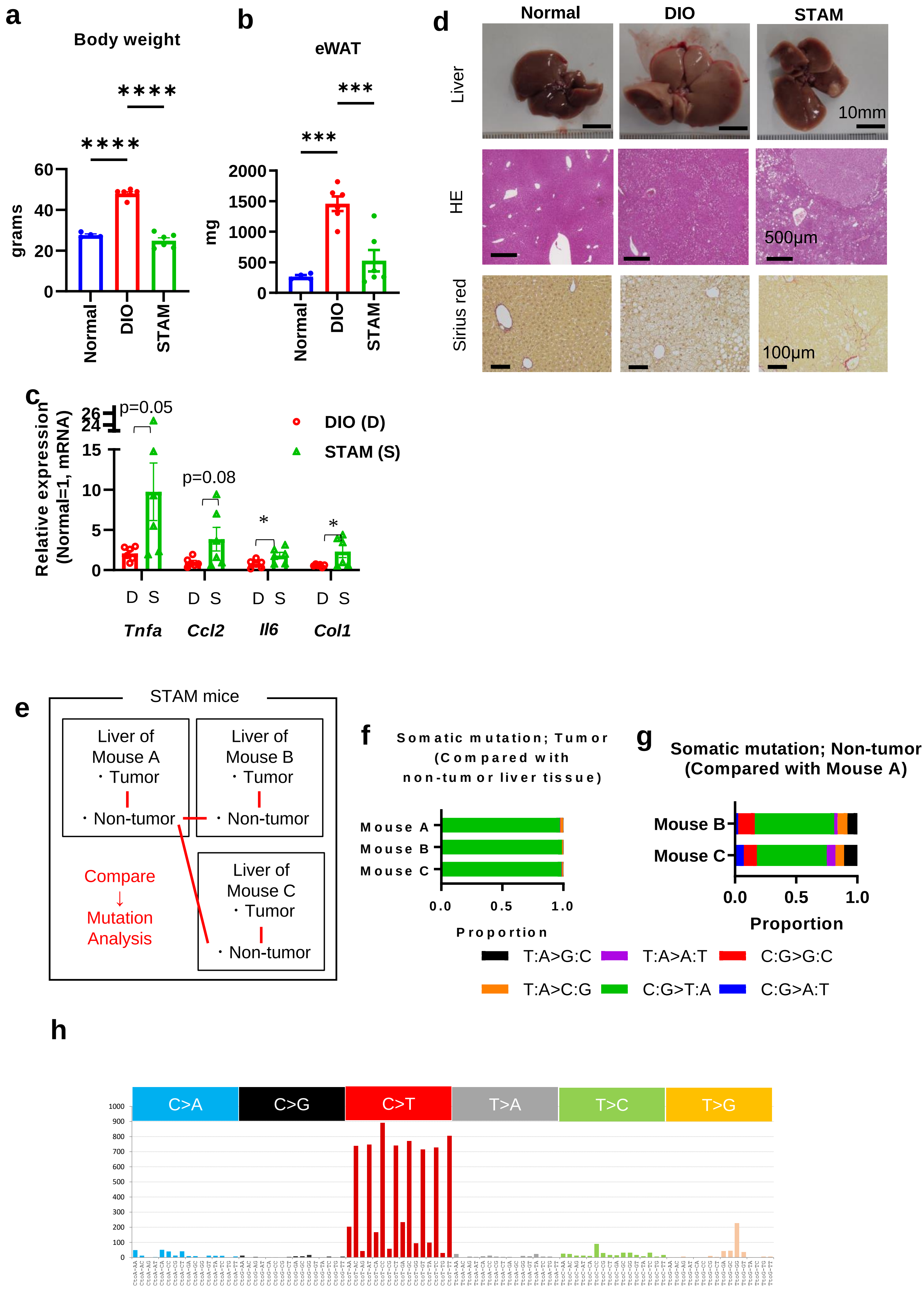

Supplementary Figure 1

Supplementary Figure 1 Related to Figure 1 Hepatic characteristics of STAM mice.

The body weight (**a**), epididymal white adipose tissue (eWAT) mass (**b**), of normal lean mice (Normal, n = 4), diet induced obese mice (DIO, n = 6), and STAM mice (n = 6). ****P* < 0.001, *****P* < 0.0001, one-way ANOVA, with Dunnnett's multiple comparisons test (**a, b**). The relative expression level of *Tnf-α, Ccl2, Il6, and Col1* determined by qPCR assay (**c**). The values are ratios to normal lean mice. DIO (n = 6) and STAM (n = 6), **P* < 0.05, 2-sided unpaired t test (**c**). The representative macroscopic and microscopic appearance (HE staining, Sirius red staining) of normal, DIO, and STAM mice (**d**). The scale bar indicates 10 mm (macroscopic appearance), 500 μm (HE staining), 100 μm (Sirius red staining). Comparison of pairs to detect somatic mutations by whole exome sequence of liver tissue (**e**). The somatic mutation type of single nucleotide in tumor tissue (**f**) compared with non-tumor part of the same mouse. Somatic mutation type of single nucleotide in non-tumor liver tissue of mouse B and C compared with that in mouse A (**g**). A 96-pattern signature from whole genome sequence of tumor samples from mouse A. The signature in C to T-XX represents clearly mutations induced by alkylating agents including streptozotocin (**h**). Values of the data are expressed as mean ± SEM (**a, b, c**). The exact P values are provided in **Supplementary Data 3**.

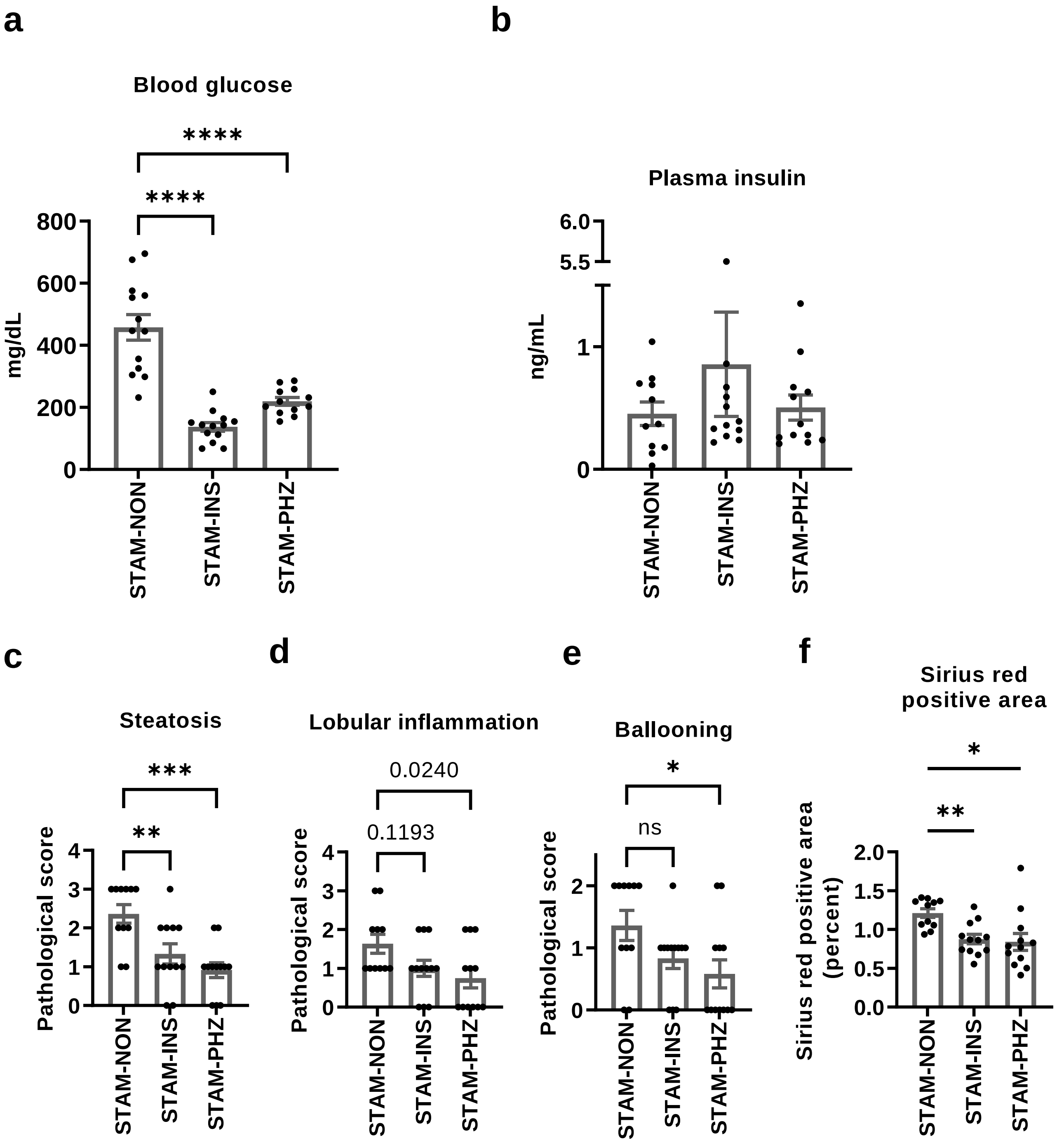

Supplementary Figure 2 Related to Figure 2 Hepatic characteristics of treated STAM mice.

Blood glucose (**a**) of STAM mice untreated (STAM-NON, n=13) or treated with insulin glargine (STAM-INS, n=13) or phlorizin (STAM-PHZ, n=12) at 20 weeks of age (**a**). ****P < 0.0001, one-way ANOVA, with Dunnett's multiple comparisons test, vs STAM-NON.

Plasma insulin (**b**), pathological grading of steatosis (**c**), lobular inflammation (**d**), ballooning (**e**), and percentage of Sirius red positive area (**f**) of STAM mice untreated (STAM-NON, n=11) or treated with insulin glargine (STAM-INS, n=12) or phlorizin (STAM-PHZ, n=12) at 20 weeks of age (**b, c, d, e, f**). *P < 0.05, **P < 0.01, ***P < 0.001, one-way ANOVA, with Dunnett's multiple comparisons test, vs STAM-NON. 2 mice in STAM-NON group and a mouse in STAM-INS group died after the day of blood glucose assay. Values of the data are expressed as mean ± SEM (**a, b, c, d, e, f**). The exact P values are provided in **Supplementary Data 3**.

Supplementary Figure 2

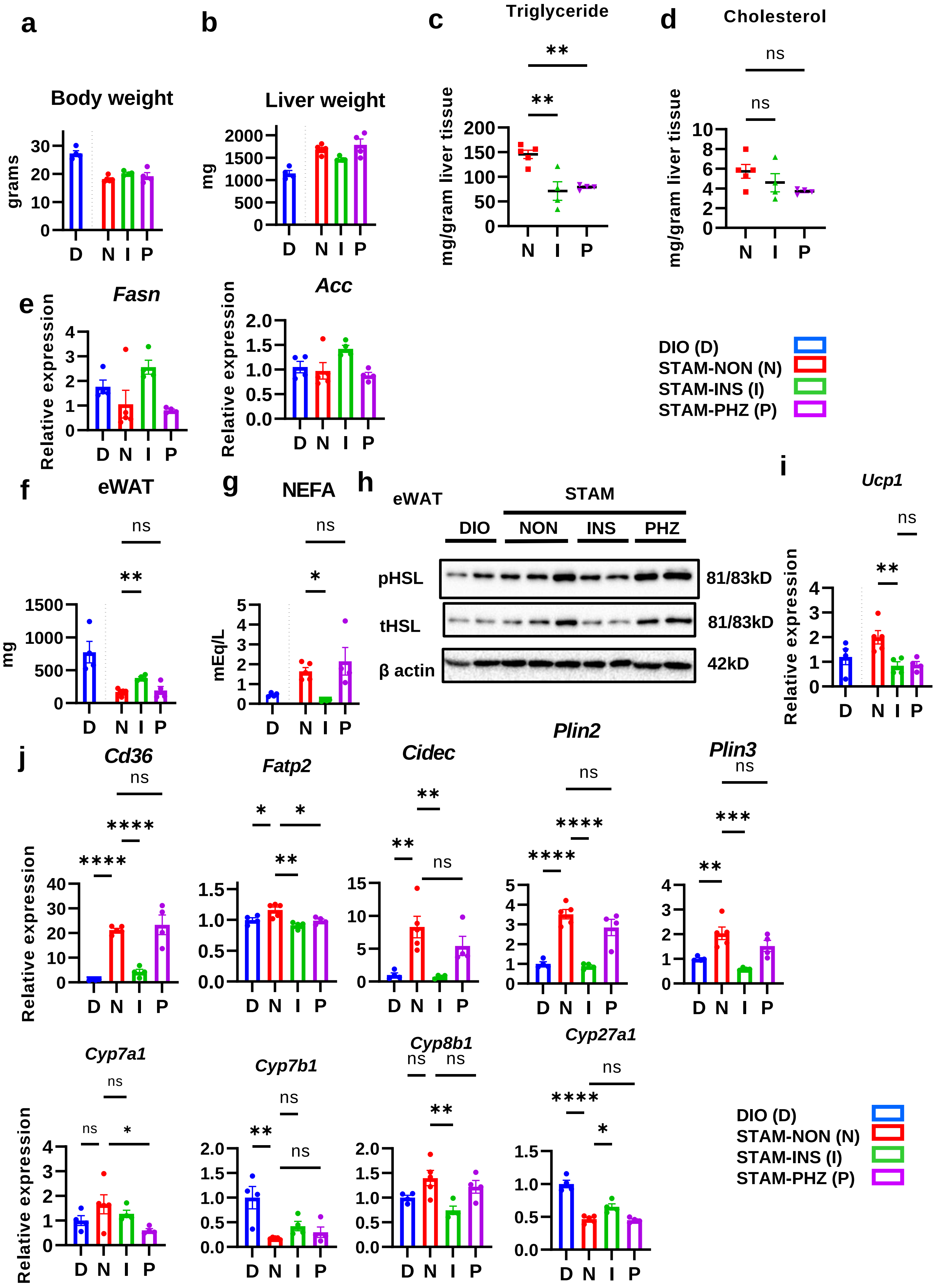

Supplementary Figure 3

Supplementary Figure 3 Related to Figure 2 Characteristics relating to hepatic steatosis in insulin-treated STAM mice and PHZ-treated STAM mice. Body weight (**a**), liver weight (**b**), hepatic triglyceride (**c**), hepatic cholesterol (**d**) at 9 weeks of age. Relative mRNA expression level of *Fasn, Acc* (**e**). Relative expression values are expressed as ratio to normal lean mice. Epididymal white adipose tissue (eWAT) weight (**f**) and plasma NEFA level (**g**). DIO mice n = 4, untreated STAM mice (STAM-NON) n = 5, insulin-treated STAM mice (STAM-INS) n = 4, and PHZ-treated STAM mice (STAM-PHZ) n = 4 at 9 weeks of age. * $P < 0.05$, ** $P < 0.01$, one-way ANOVA with Dunnett's multiple comparisons test vs STAM-NON with STAM-INS and STAM-PHZ (**a, b, c, d, e, f, g**). Western blotting by eWAT lysate of STAM mice at 9 weeks of age (**h**). The experiments were repeated independently at least twice. Anti-pHSL, anti-tHSL, anti-β-actin antibodies were used as primary antibodies to detect each molecule. Relative expression level of *Ucp1* in eWAT (**i**), and relative expression of *Cidec, Plin2, Plin3, Cd36, Fatp2, Igf-1, Igf-1r, Cyp7a1, Cyp7b1, Cyp8ba, Cyp27a1* in liver (**j**), of DIO mice n = 4, STAM-NON n = 5, STAM-INS n = 4, and STAM-PHZ n = 4 at 9 weeks of age. * $P < 0.05$, ** $P < 0.01$, *** $P < 0.001$, **** $P < 0.0001$, one-way ANOVA with Dunnett's multiple comparisons test vs STAM-NON, with STAM-INS and STAM-PHZ (**i**), or with DIO, STAM-INS and STAM-PHZ (**j**). Values of the data are expressed as mean ± SEM (**a, b, d, c, e, f, g, i, j**). The exact P values are provided in **Supplementary Data 3**.

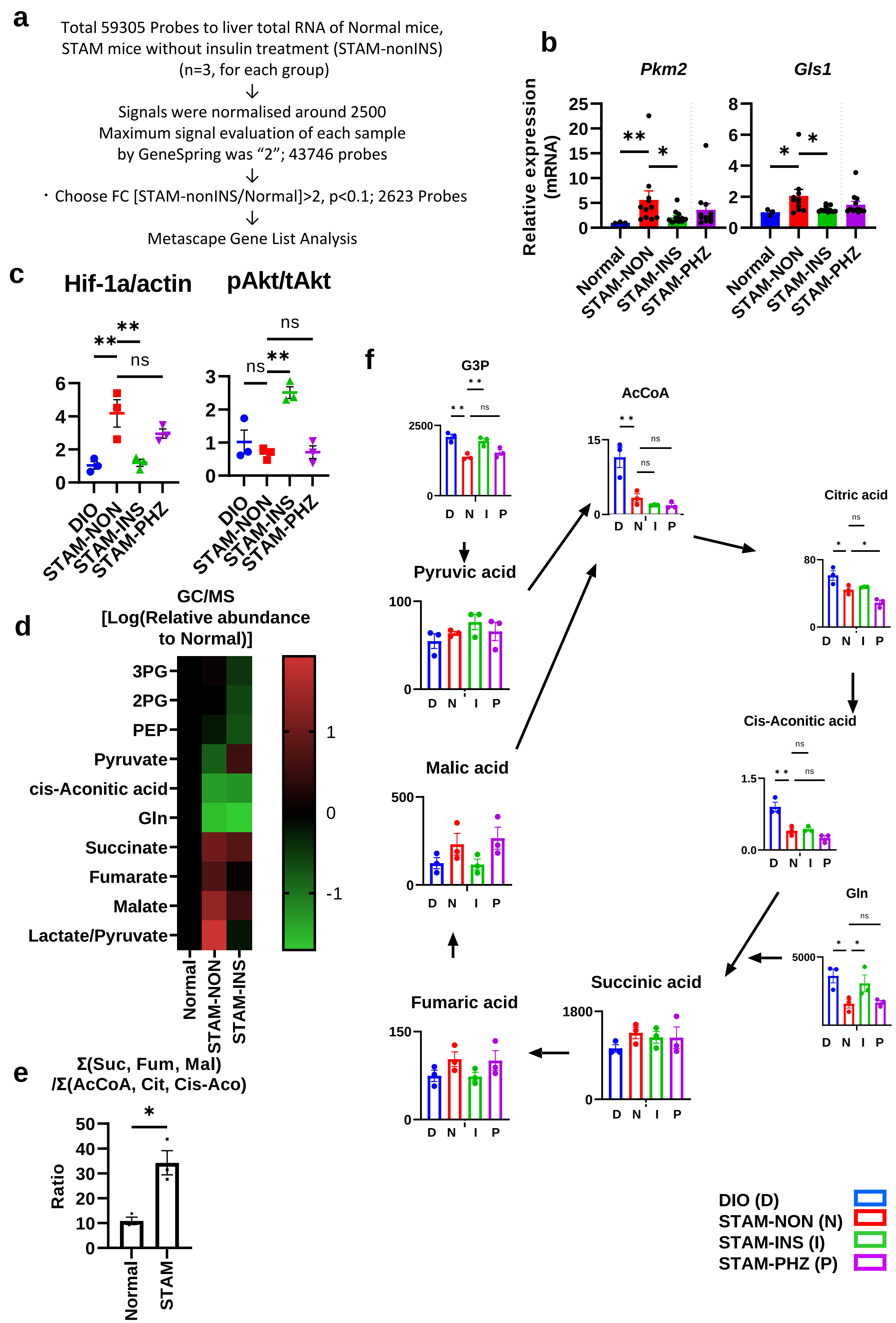

Supplementary Figure 4

Supplementary Figure 4 Related to Figure 3 Characteristics relating to hepatic metabolomic changes in insulin-treated STAM mice.
The process of gene ontology analysis by the list of upregulated genes in STAM mice in microarray analysis (**a**). Relative expression level of *Pkm2, Gls1* in 20-week-old STAM mice liver (**b**). Normal (n = 3), non-treated STAM mice (STAM-NON, n = 11), insulin-treated STAM mice (STAM-INS, n = 12), and phlorizin-treated STAM mice (STAM-PHZ, n = 12), *P < 0.05, **P < 0.01, one-way ANOVA, with Dunnett's multiple comparisons test in Normal, STAM-NON, STAM-INS (**b**). Quantified densitometric analysis of Hif-1a and pAkt for **Fig.3b**, normalized by actin and tAkt, respectively (**c**). n = 3, **P < 0.01, *P < 0.05, Dunnett's multiple comparisons test (**c**). CE-TOF/MS analysis of the members of the glycolysis pathway and the TCA cycle. Heat map is generated from logarithm of relative abundance ratio to normal mice (**d**). The ratio of the sum of the latter half (succinate, fumarate, malate) to that of the former half (acetyl CoA, citrate, cis-aconitic acid) of TCA cycle of 20-week-old STAM mice (**e**). n = 3, *P < 0.05, 2-sided unpaired t-test (**e**). Hepatic accumulation of members in the glycolysis pathway and the TCA cycle. The amount of glycerardehyde-3-phosphate (G3P), pyruvic acid, acetyl CoA, citric acid, cis-aconitic acid, glutamine (Gln), 2-HG, succinic acid, fumaric acid and malic acid were determined by CE-TOF/MS using liver tissue of 9-week-old STAM mice (**f**). DIO mice n = 3, STAM-NON n = 3, STAM-INS n = 3, and STAM-PHZ n = 3 at 9 weeks of age. *P < 0.05, **P < 0.01, ***P < 0.001, one-way ANOVA, with Dunnett's multiple comparisons test (except for Gln) and Two-stage linear step-up procedure of Benjamini, Krieger and Yekutieli (Gln). Values of the data are expressed as mean ± SEM (**b, c, e, f**). The exact P values are provided in **Supplementary Data 3**.

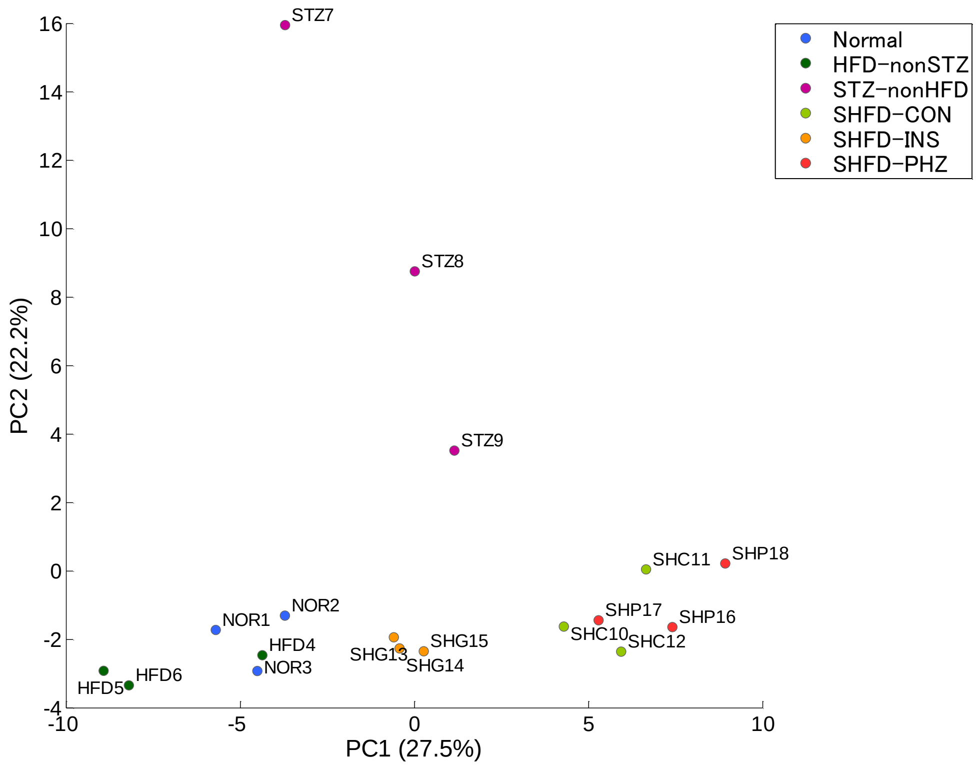

Supplementary Figure 5, Related to Figure 3 Primary component analysis from CE-TOF/MS analysis in liver of STAM mice with insulin or phlorizin treatment.

Primary component analysis was conducted by the software invented by human metabolome technologies. The liver tissue samples from mice in their 9 weeks of age were under analysis. SHFD-CON, for control STAM mice. SHFD-INS, for STAM mice with insulin treatment. SHFD-PHZ, for STAM mice with phlorizin treatment. The following groups were also included in the analysis along with drug treatment to evaluate the influence of feeding and streptozotocin (STZ) treatment in this model mice. Normal, for mice fed with normal chow. HFD-nonSTZ, for mice fed with high fat diet (HFD) and without treatment of STZ on birth. STZ-nonHFD, for mice fed with normal chow and treated with STZ on birth. Each group includes 3 samples. The exact P values are provided in **Supplementary Data 3**.

Supplementary Figure 5

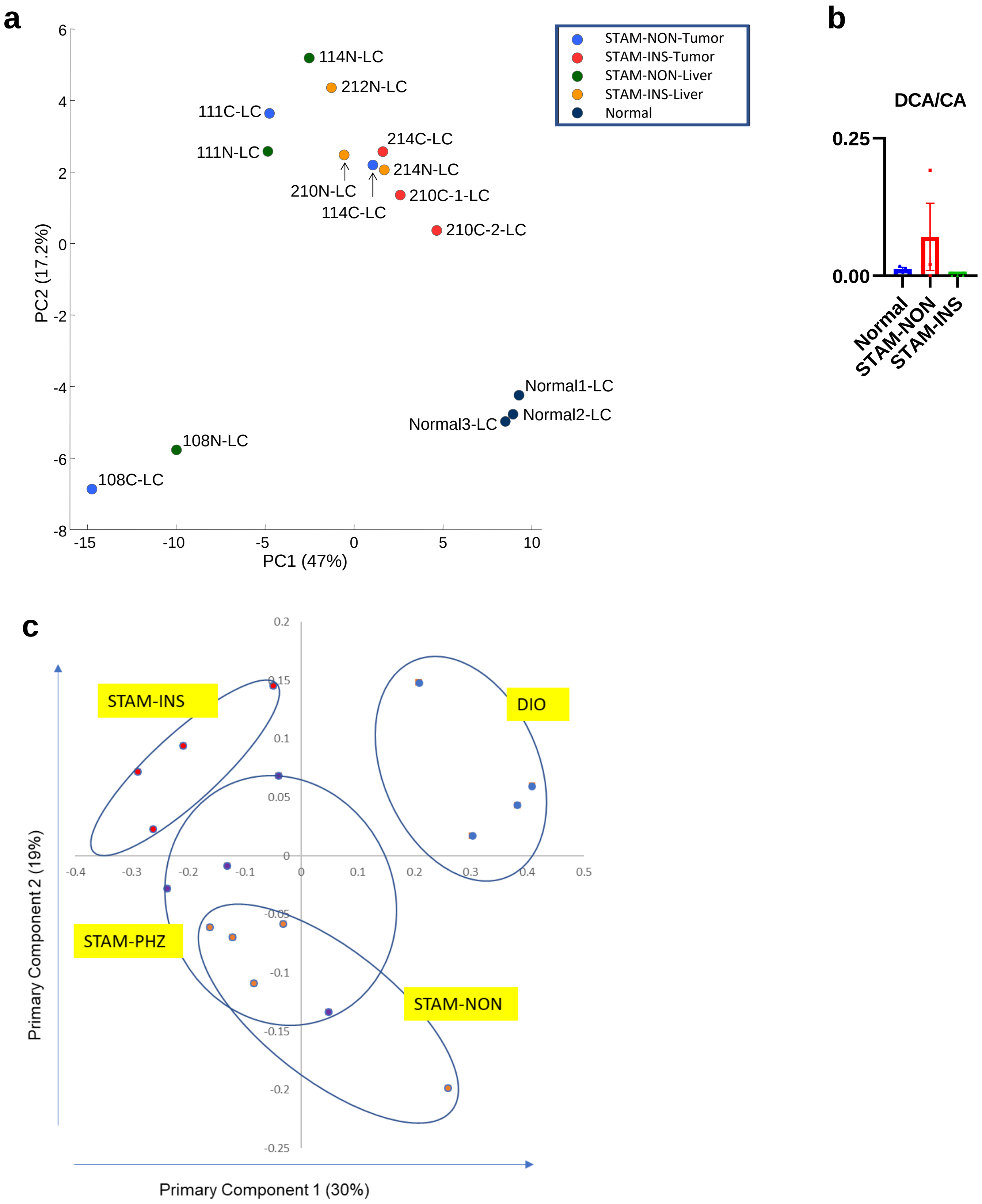

Supplementary Figure 6, Related to Figure 4 Characteristics relating to hepatic metabolomic and fecal metagenomic changes in insulin-treated STAM mice.

Primary component analysis from LC-MS analysis was conducted by the software invented by human metabolome technologies. LC-MS analysis was conducted from tumor or non-tumor liver sample of 20-week-old STAM mice. STAM-NON-Tumor, for tumor samples from STAM mice without treatment. STAM-INS-Tumor, for tumor samples from insulin-treated STAM mice. STAM-NON-Liver, for non-tumor liver tissue from STAM mice without treatment. STAM-INS-Liver, for non-tumor liver tissue from insulin-treated STAM mice. Normal, for normal-chow-fed mice without STZ-treatment on birth. (**a**) Each group includes 3 samples.

The ratio of deoxycholic acid (DCA) to cholic acid (CA) in liver (**b**). Values of the data are expressed as mean ± SEM (**b**).

Primary component analysis (PCoA) of 16S metagenomics in 9-week-old STAM mice (**c**). DIO mice n = 4, non-treated STAM mice (STAM-NON) n = 5, insulin-treated STAM mice (STAM-INS) n = 4, and PHZ-treated STAM mice (STAM-PHZ) n = 4 at 9 weeks of age. Values of the data are expressed as mean ± SEM. The exact P values are provided in **Supplementary Data 3**.

Supplementary Figure 6

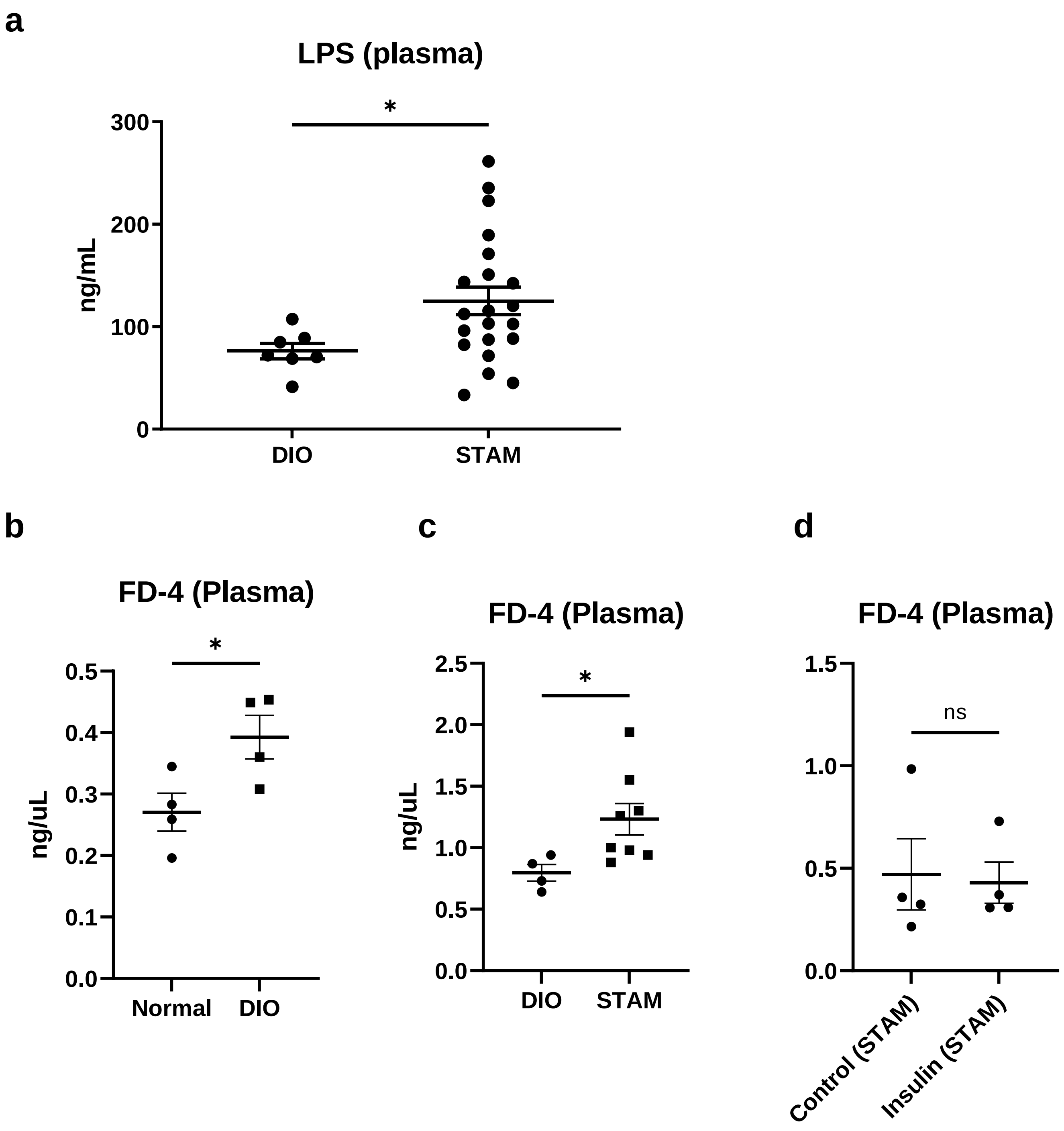

Supplementary Figure 7, Related to Figure 4 Physical barrier dysfunction in STAM mice. Lipopolysaccharide (LPS) concentration in plasma (**a**). DIO mice (n = 7), STAM mice (n = 21). The concentration of fluorescein isothiocyanate–dextran – 4kDa (FD-4) in plasma. The comparison was conducted between normal mice (n = 4) and diet-induced-obese (DIO) mice (n = 4) (**b**), between DIO mice (n = 4) and STAM mice (n = 8) (**c**), and between STAM mice without treatment (n = 4) and insulin-treated STAM mice (n = 4) (**d**). Values of the data are expressed as mean ± SEM (**a, b, c, d**). * $P < 0.05$, 2-sided Mann-Whitney's u test (**a**), and 2-sided unpaired t test (**b, c, d**). The exact P values are provided in **Supplementary Data 3**.

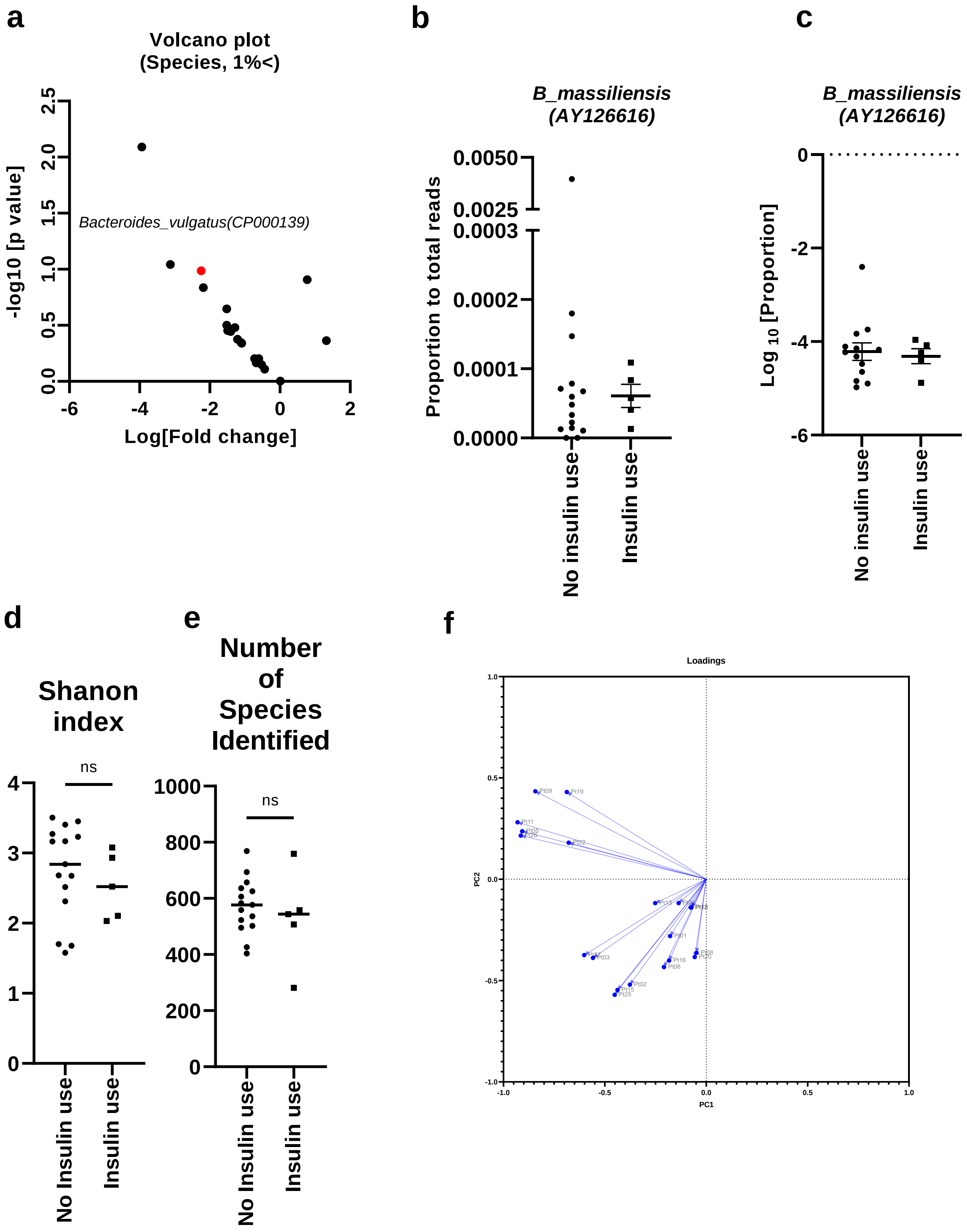

Supplementary Figure 8, Related to Figure 5 16S metagenomic signature alteration by insulin use in NASH patients with diabetes

Volcano plot of proportion to total reads of fold change by insulin treatment and *P* value by ANCOM-BC2 test between two groups (**a**). Species shown here are selected by average proportion over 1% in total reads in the group without insulin treatment. (See also **Supplementary Table 7**.)

Proportion of *Bacteroides massiliensis* (AY126616) (**b**) in non-insulin-treated (no insulin use, n = 15) and insulin-treated NASH patients (insulin use, n = 5). The logarithm 10 of proportion of *Bacteroides massiliensis* (AY126616) (**c**) in non-insulin-treated (no insulin use, n = 13) and insulin-treated NASH patients (insulin use, n = 5). 2 samples in non-insulin-treated group were excluded because of no detection of this bacterium by this method. Values of the data are expressed as mean ± SEM (**b, c**).

Shannon index (**d**), the number of identified species (**e**), and loading data from primary component analysis (**f**) in non-insulin-treated (no insulin use, n = 15) and insulin-treated NASH patients (insulin use, n = 5), compared by 2-sided unpaired t test. Values of the data are expressed as mean ± SEM (**b, c**). Source data are provided as a **Supplementary Data 3**.

Supplementary Figure 8

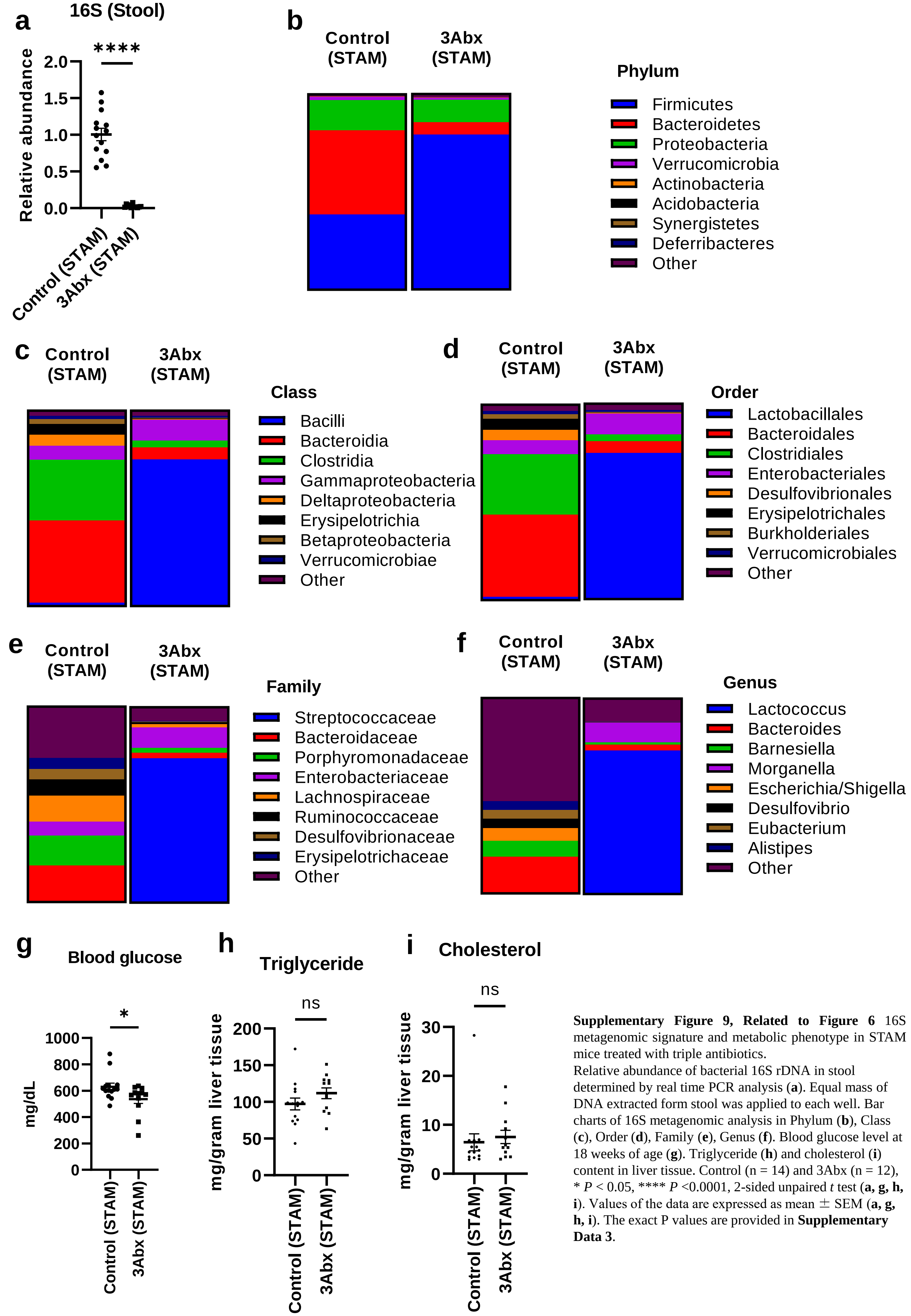

Supplementary Figure 9, Related to Figure 6 16S metagenomic signature and metabolic phenotype in STAM mice treated with triple antibiotics.
Relative abundance of bacterial 16S rDNA in stool determined by real time PCR analysis (**a**). Equal mass of DNA extracted form stool was applied to each well. Bar charts of 16S metagenomic analysis in Phylum (**b**), Class (**c**), Order (**d**), Family (**e**), Genus (**f**). Blood glucose level at 18 weeks of age (**g**). Triglyceride (**h**) and cholesterol (**i**) content in liver tissue. Control (n = 14) and 3Abx (n = 12), * $P < 0.05$, **** $P < 0.0001$, 2-sided unpaired *t* test (**a, g, h, i**). Values of the data are expressed as mean ± SEM (**a, g, h, i**). The exact P values are provided in **Supplementary Data 3**.

Supplementary Figure 9

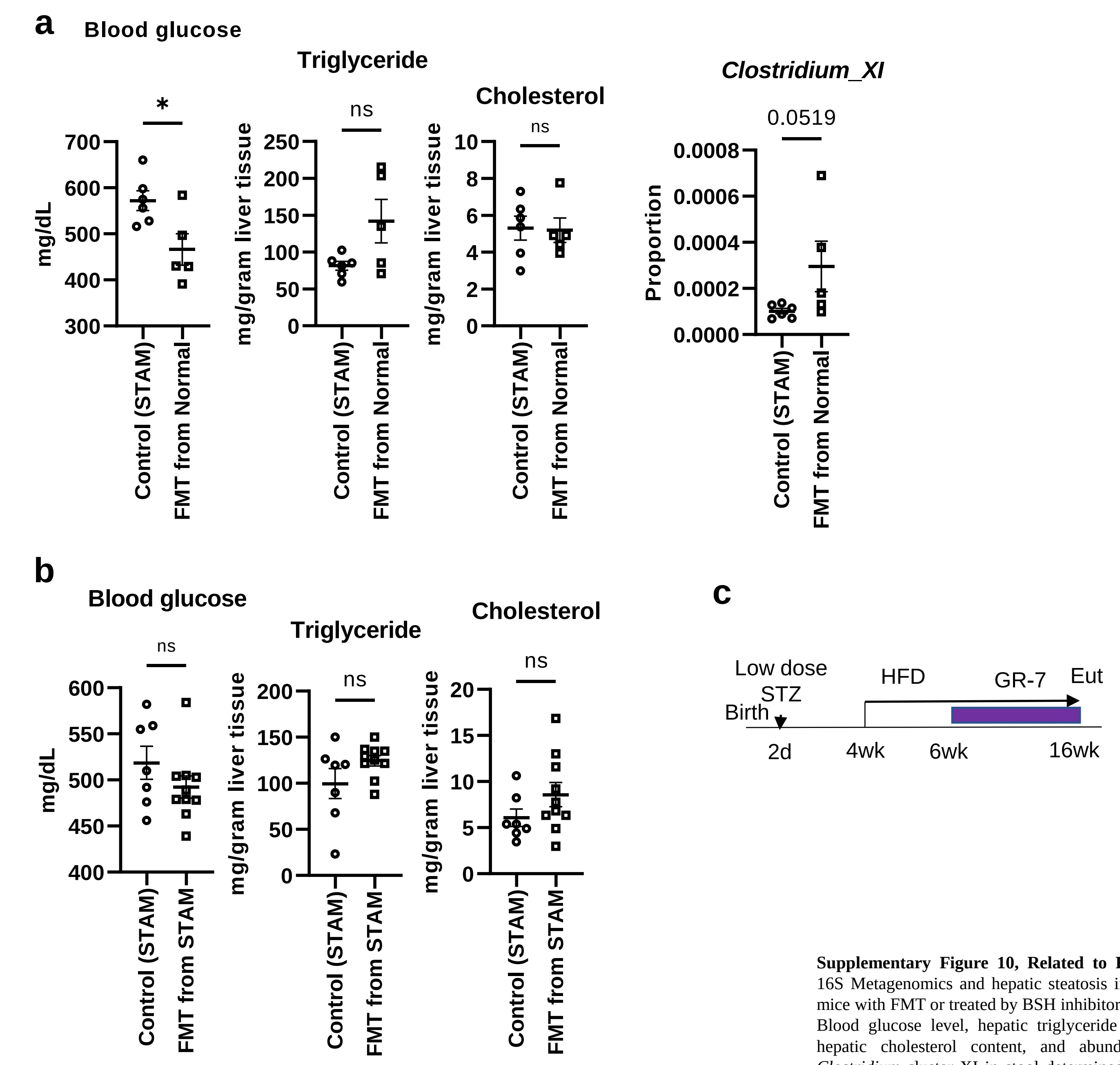

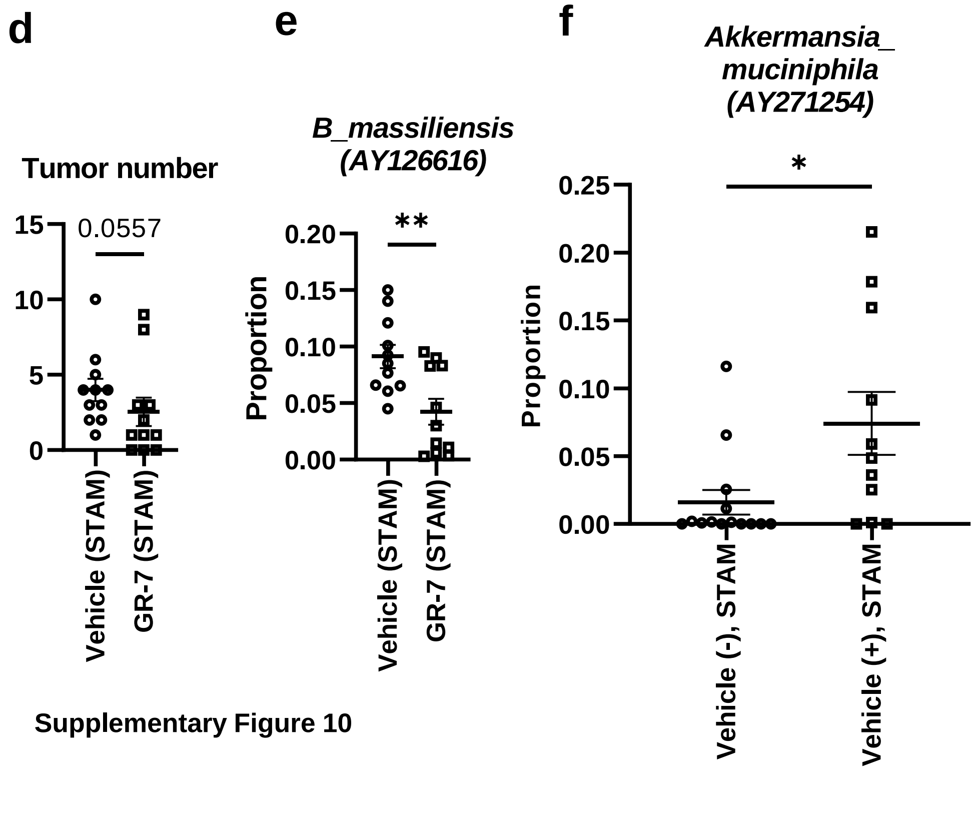

Supplementary Figure 10

Supplementary Figure 10, Related to Figure 6 16S Metagenomics and hepatic steatosis in STAM mice with FMT or treated by BSH inhibitor, GR-7.
Blood glucose level, hepatic triglyceride content, hepatic cholesterol content, and abundance of *Clostridium* cluster XI in stool determined by 16S metagenomics in STAM mice with fecal microbiota transplantation (FMT) treatment from normal mice (**a**). Blood glucose level, hepatic triglyceride content, and cholesterol content in STAM mice with FMT from STAM mice (**b**). For FMT experiment from normal mice, Control (STAM), n = 6. FMT from normal (STAM), n = 5 (**a**). For FMT experiment from STAM mice, Control (STAM), n = 7. FMT from STAM (STAM), n = 10 (**b**). * $P < 0.05$, 2-sided unpaired t test (**a, b**).
The experiment protocol for adminis”rati’n of GR-7 (Gut restricted-7) to STAM mice(**c**). Tumor number (**d**), read proportion of *Bacteroides massiliensis* (AY126616) (**e**), Control (STAM), n = 11, GR-7 (STAM), n = 11. Comparison of read proportion of *Akkermansia muciniphila* (AY271254) between STAM mice without treatment and those with vehicle treatment (n = 11) (**f**). The former group is identical to control against triple antibiotics-treated STAM mice (n = 14). * $P < 0.05$, ** $P < 0.01$, 2-sided unpaired t test (**d, e, f**).
Values of the data are expressed as mean ± SEM (**a, b, d, e, f**). The exact P values are provided in **Supplementary Data 3**.

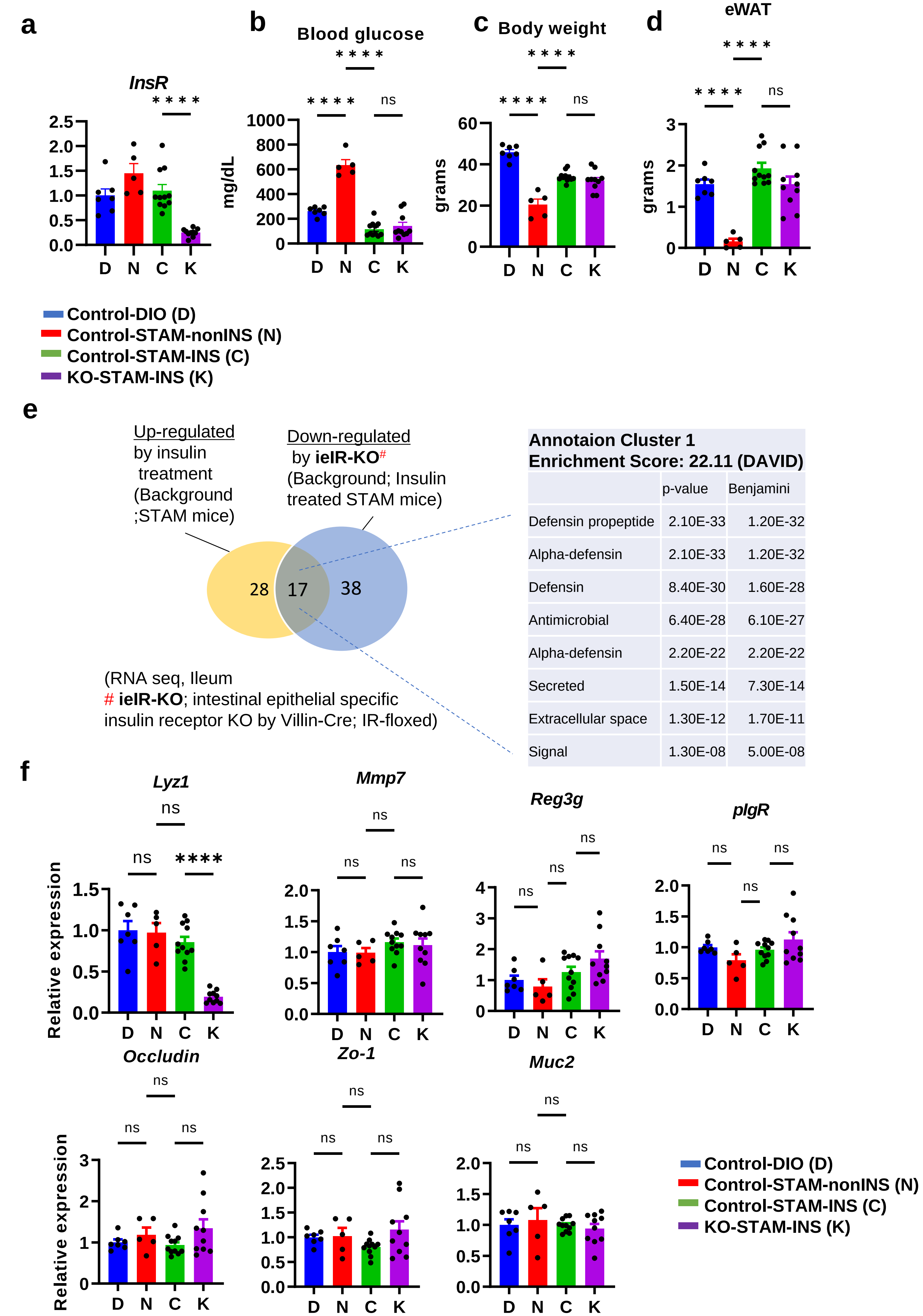

Supplementary Figure 11

Supplementary Figure 11, Related to Figure 7 Gross phenotype and ileal phenotype of insulin-treated ieIRKO-STAM mice. Ileal relative mRNA expression of *InsR* **(a)**, blood glucose (**b**), body weight (**c**), eWAT weight (**d**) of 20-week-old, insulin-treated ieIRKO-STAM mice. Control-DIO n = 7, non-insulin-treated control STAM mice (Control-STAM-nonINS) n = 5, insulin-treated control STAM mice (Control-STAM-INS) n = 11, insulin-treated intestinal epithelial insulin receptor knock out mice (ieIRKO-STAM-INS) n = 10. *****P* < 0.0001, qPCR analysis with one-way ANOVA, Šídák's multiple comparisons test between adjacent two groups.

Venn diagram of RNA sequence analysis in ileal whole wall of each group (**e**). Yellow circle contains genes significantly up-regulated by insulin treatment in STAM mice, and blue circle contains genes significantly down-regulated by intestinal epithelial insulin receptor knock-out in insulin-treated STAM mice (Control-STAM-nonINS n = 4, Control-STAM-INS n = 6 vs ieIRKO-STAM-INS n = 6, threshold FC, 2 and 0.5, respectively, FDR *P* value, < 0.05; max group mean read number, > 300) (**e**). Enrichment analysis performed by DAVID software (**e**, right). Ileal relative mRNA expression of *Lyz1, Mmp7, Reg3g, pIgR, Occludin, Zo1,* and *Muc2* (**f**). Control-DIO n = 7, Control-STAM-nonINS n = 5, Control-STAM-INS n = 11 vs ieIRKO-STAM-INS n = 10, *****P* < 0.0001, qPCR analysis with one-way ANOVA, Šídák's multiple comparisons test between adjacent two groups (**f**). Values of the data are expressed as mean ± SEM (**a, b, c, d, f**). The exact P values are provided in **Supplementary Data 3**.

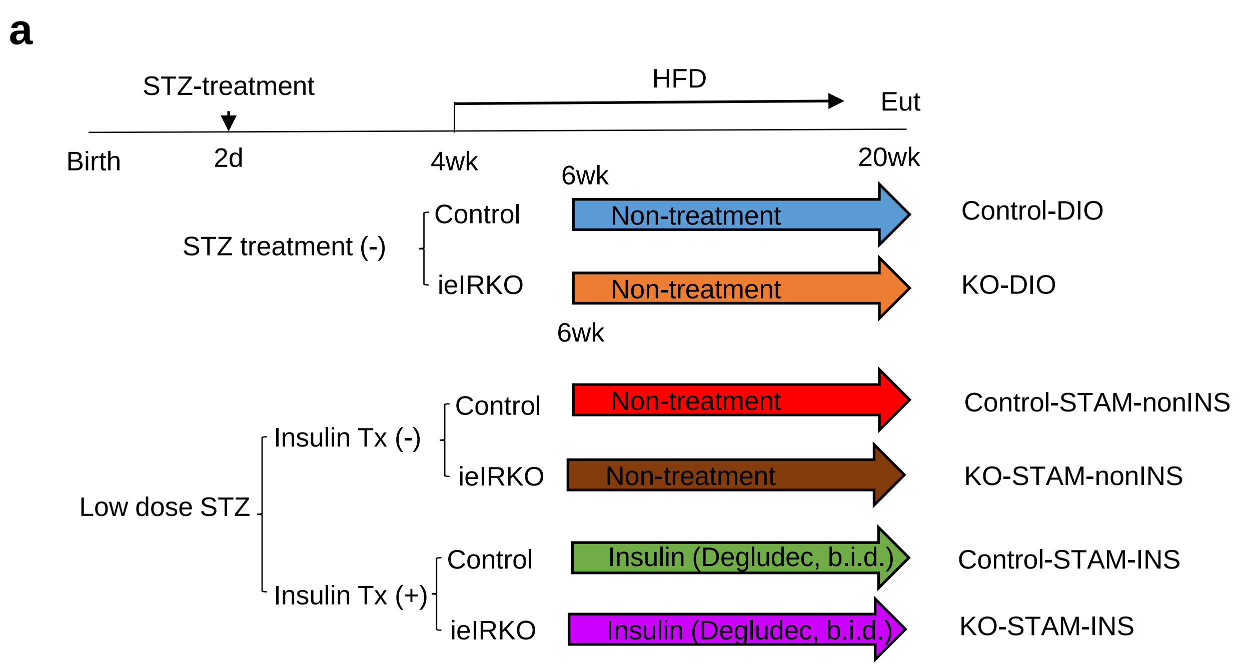

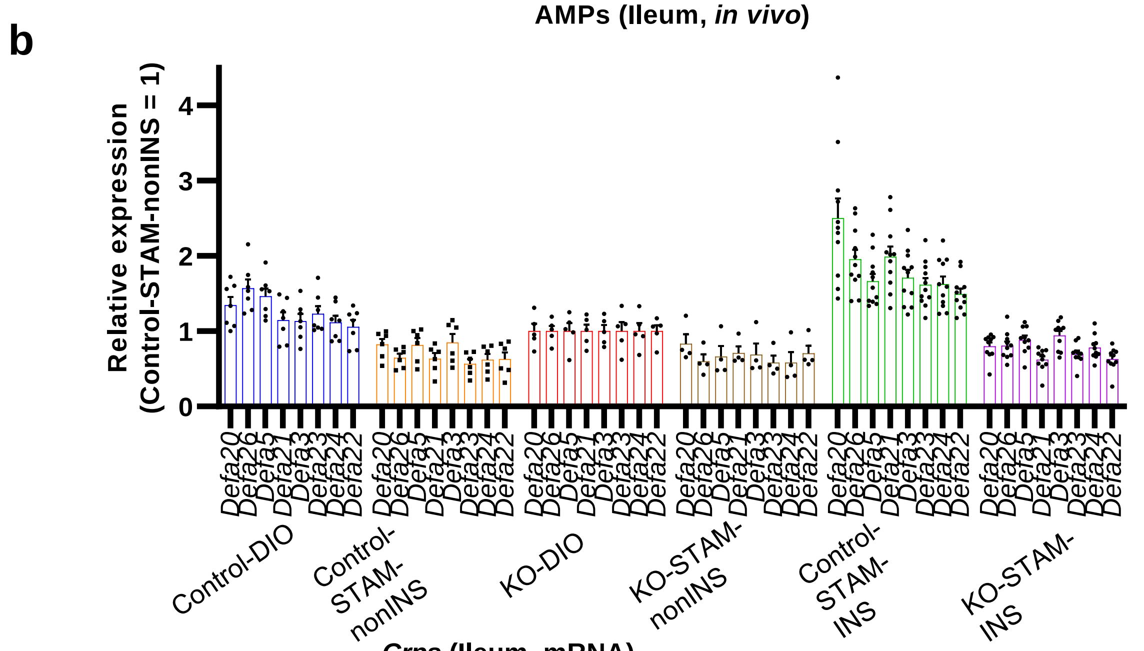

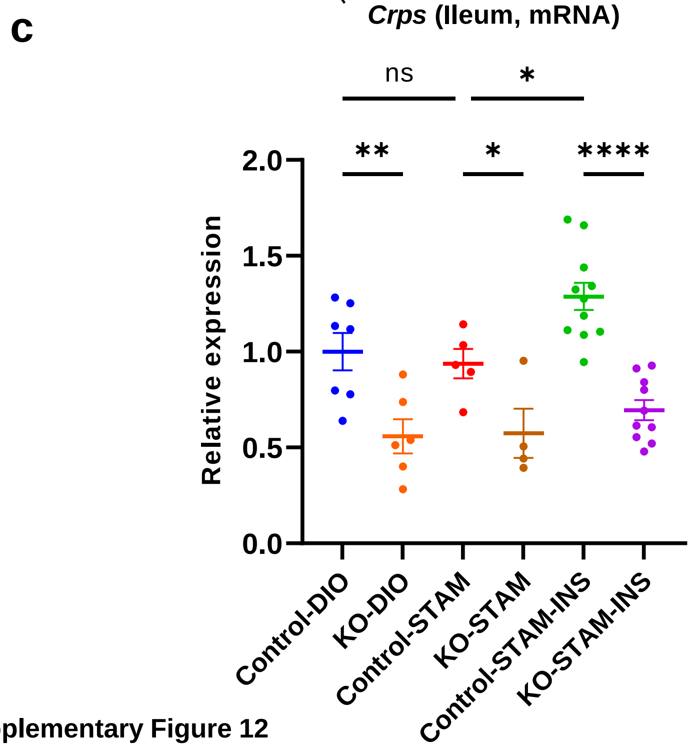

Supplementary Figure 12, Related to Figure 7 The expression level of antimicrobial peptides (AMPs) in ileum in ieIRKO mice in three conditions.

Comparison of expression level between intestinal epithelial insulin receptor knock out mice (ieIRKO) and control mice in the following conditions, DIO, STAM without treatment, insulin-treated STAM (**a**). Target genes are *Defa 3, Defa 5, Defa 20, Defa 21, Defa 22, Defa 23, Defa 24, Defa 26* (**b**), and *Crps* (**c**). Control-DIO n = 7, intestinal epithelial insulin receptor knock out DIO mice (KO-DIO) n = 6, non-insulin-treated control STAM mice (Control-STAM-nonINS) n = 5, non-insulin-treated intestinal epithelial insulin receptor knock out mice (KO-STAM-nonINS) n = 4, insulin-treated control STAM mice (Control-STAM-INS) n = 11 vs insulin-treated intestinal epithelial insulin receptor knock out mice (KO-STAM-INS) n = 10, **P* < 0.05, ***P* < 0.01, *****P* < 0.0001, qPCR analysis with one-way ANOVA, Holm-Šídák's multiple comparisons test (**b, c**). Values of the data are expressed as mean ± SEM (**b, c**). The exact P values are provided in **Supplementary Data 3**.

Supplementary Figure 12

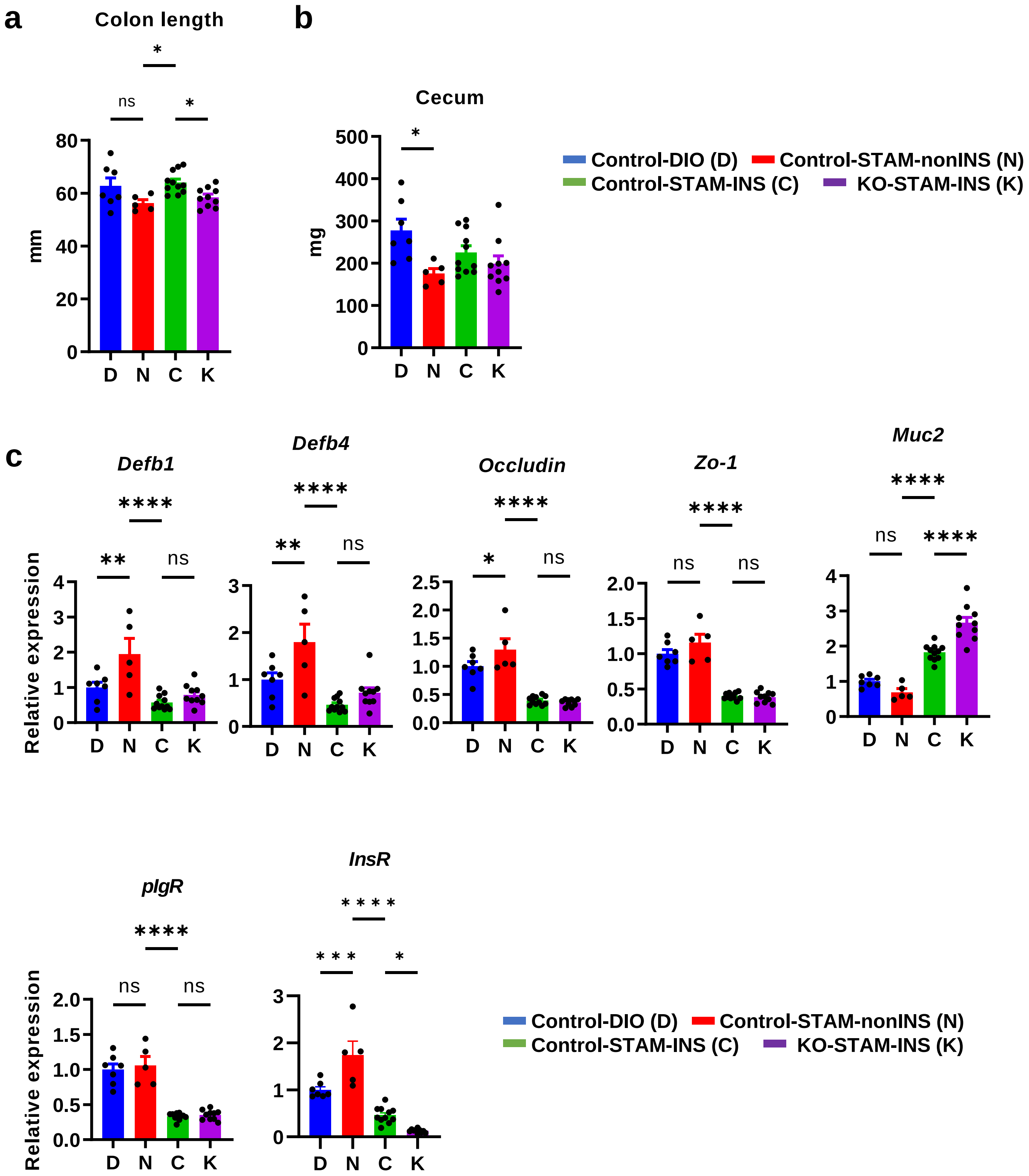

Supplementary Figure 13, Related to Figure 7 Colon phenotype of insulin-treated ieIRKO-STAM mice.
Colon length (**a**), cecal weight (**b**). Relative expression level by qPCR analysis of *Defb1, Defb4, Occludin, Zo1, Muc2, pIgR,* and *InsR* (**c**). Control-DIO n = 7, non-insulin treated control STAM mice (Control-STAM-nonINS) n = 5, insulin-treated control STAM mice (Control-STAM-INS) n = 11, insulin-treated intestinal epithelial insulin receptor knock out mice (ieIRKO-STAM-INS) n = 10. *$P < 0.05$, **$P < 0.01$, ***$P < 0.001$, ****$P < 0.0001$, one-way ANOVA, Šídák's multiple comparisons test between adjacent two groups. Values of the data are expressed as mean ± SEM (**a, b, c**). The exact P values are provided in **Supplementary Data 3**.

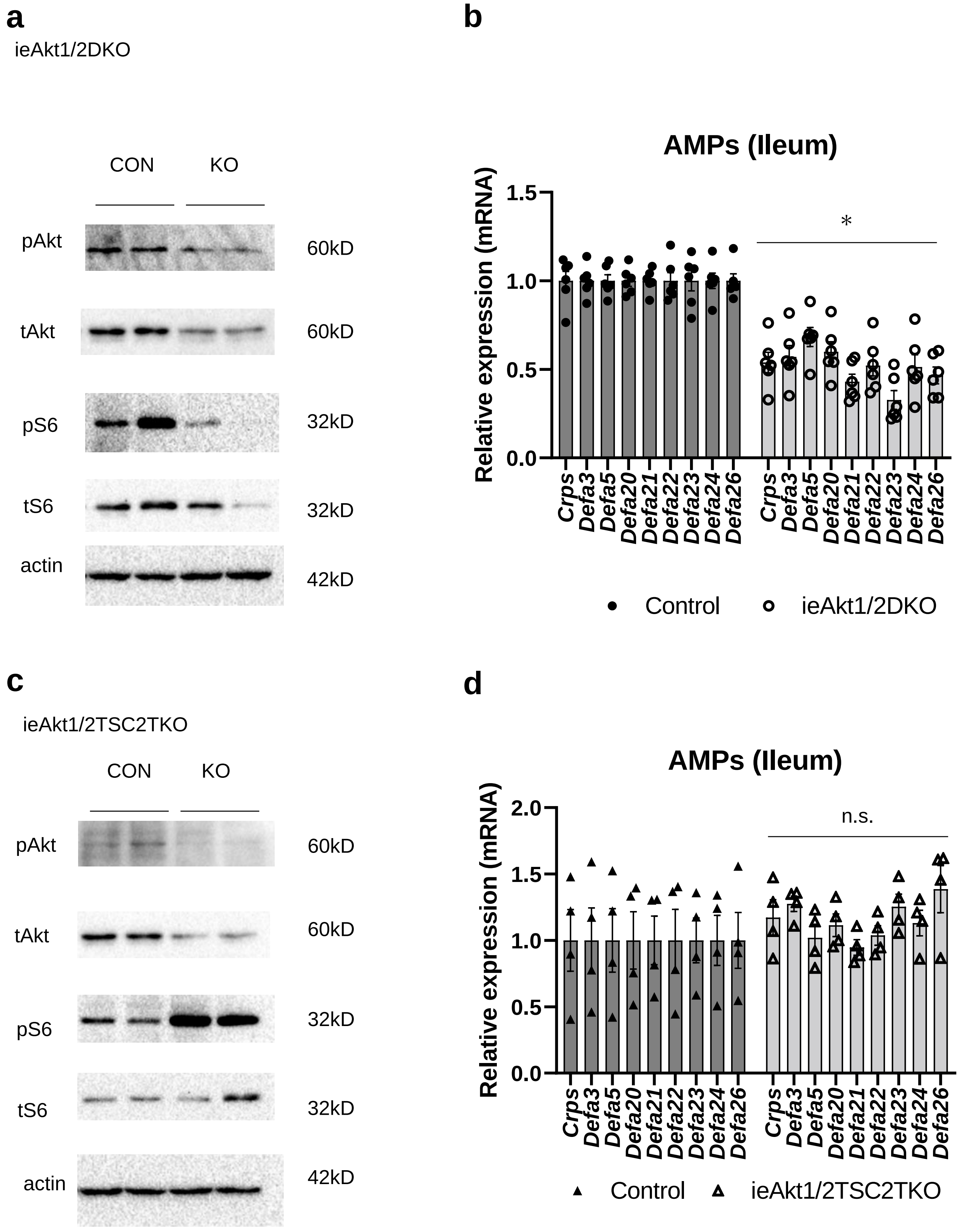

Supplementary Figure 14, Related to Figure 7 The causal relevance between intestinal epithelial Akt/TSC2 signal and AMPs expression *in vivo*. Western blotting analysis of ileal tissue in intestinal epithelium-specific Akt1 and Akt2 double knock out mice (ieAkt1/2DKO mice) at 8 weeks of age. Mice were fasted overnight and injected of Humarin R ® 2u/head via inferior vena cava. Ileum was rapidly excised and snap frozen tissue sample was applied to western blotting (n = 2). The probe antibody was pAkt (S473), total Akt, pS6, total S6, beta actin (**a**). The relative expression level of AMPs in ieAkt1/2DKO mice and littermate control mice (**b**). (n = 6, * $P < 0.05$, 2-sided unpaired t test against littermate control, **b**)
Western blotting analysis of ileal tissue in intestinal epithelium-specific Akt1, Akt2 and TSC2 triple knock out mice (ieAkt1/2TSC2TKO mice, n = 2, **c**) at 8 weeks of age. The assay procedure was the same as in ieAkt1/2DKO mice. The relative expression level of AMPs in ieAkt1/2TSC2TKO mice (n = 4, * $P < 0.05$, 2-sided unpaired t test against littermate control, **d**). Values of the data are expressed as mean ± SEM (**b, d**). The exact P values are provided in **Supplementary Data 3**.

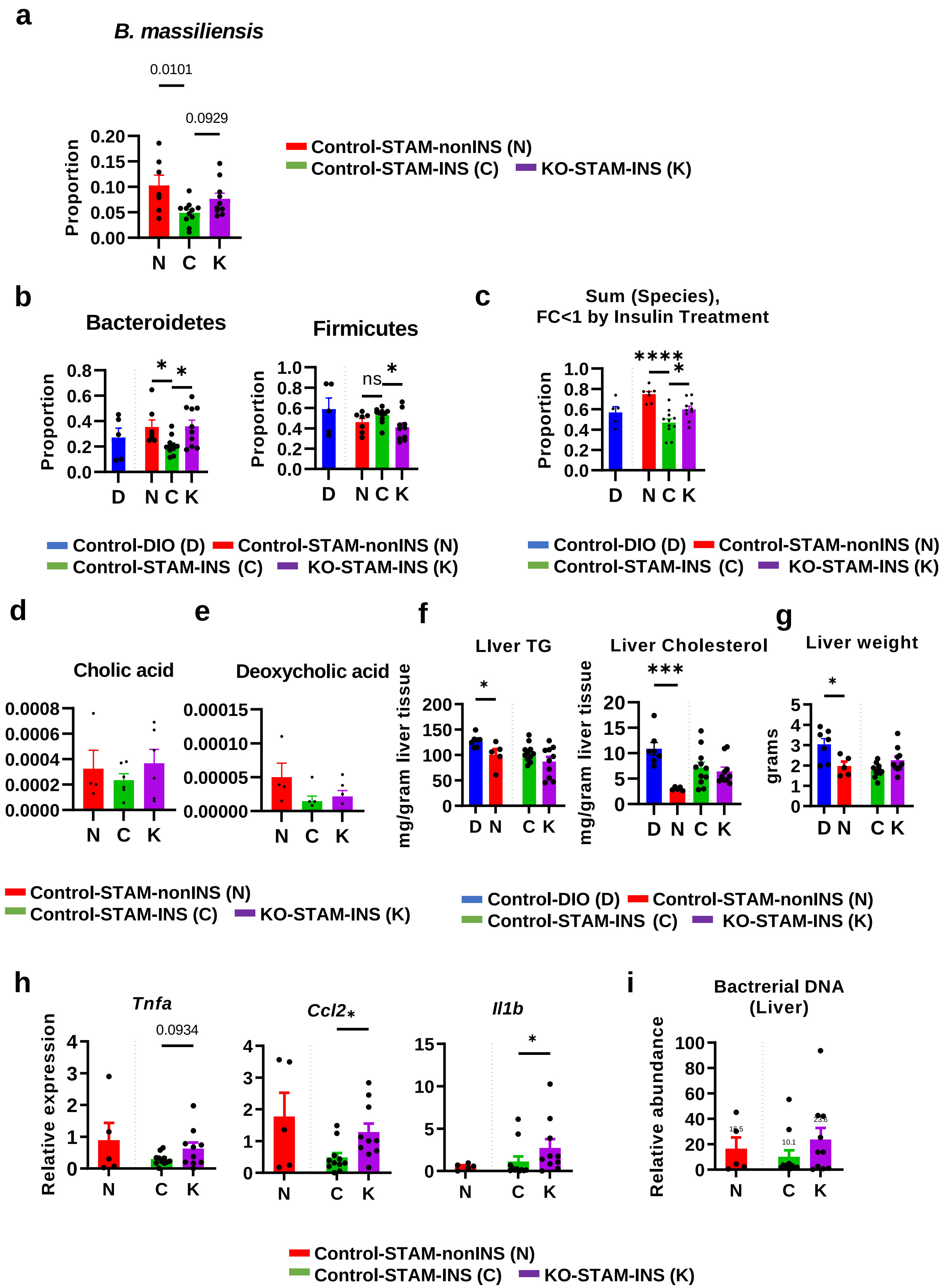

Supplementary Figure 15

Supplementary Figure 15, Related to Figure 7 Gut floral phenotype and liver phenotype of insulin-treated ieIRKO-STAM mice.

Read proportion of *Bacteroides massiliensis* (**a**). Non-insulin-treated control STAM mice (Control-STAM-nonINS) n = 7, insulin-treated control STAM mice (Control-STAM-INS) n = 11, insulin-treated intestinal epithelial insulin receptor knock-out STAM mice (KO-STAM-INS) n = 10. *P < 0.05, one-way ANOVA, Holm-Šídák's multiple comparisons test between adjacent two groups. Phylum level analyses for Bacteroides and Firmicutes (**b**). DNA was extracted from collectable fresh fecal samples from each mouse. Control-DIO (n = 5) was presented as reference. Statistical analysis was performed by Control-STAM-nonINS n = 7, Control-STAM-INS n = 11, KO-STAM-INS n = 10. *P < 0.05, one-way ANOVA, Šídák's multiple comparisons test between adjacent two groups. Sum of the proportion of species, whose proportion was suppressed by insulin treatment. The criteria and selected species are indicated in Method section (**c**). Statistical analysis was performed as in **Supplementary Fig. 15b**. Šídák's multiple comparisons test between adjacent two groups, *P < 0.05, ****P < 0.0001 (**c**).

Comparison of cholic acid (**d**), deoxycholic acid (**e**) in liver of control-STAM-nonINS, control-STAM-INS, KO-STAM-INS

Liver triglyceride content, cholesterol content (**f**) and liver weight (**g**) of 20-week-old, insulin-treated KO-STAM mice. Control-DIO n = 7, Control-STAM-nonINS n = 5, Control-STAM-INS n = 11, KO-STAM-INS n = 10. *P < 0.05, 2-sided unpaired t test (**f, g**).

Hepatic relative mRNA expression of *Tnfa, Ccl2,* and *Il1b* (**h**). Hepatic relative abundance of bacterial 16S rDNA determined by qPCR (**i**). Control-STAM-nonINS n = 5, Control-STAM-INS n = 11, KO-STAM-INS n = 10. Each P value and *P < 0.05 are analyzed by 2-sided unpaired t test except for *Il1b* (**h**). 2-sided Mann-Whitney's *u* test was applied for *Il1b* (**h**). Values of the data are expressed as mean ± SEM (**a, b, c, d, e, f, g, h, i**). The exact P values are provided in **Supplementary Data 3**.

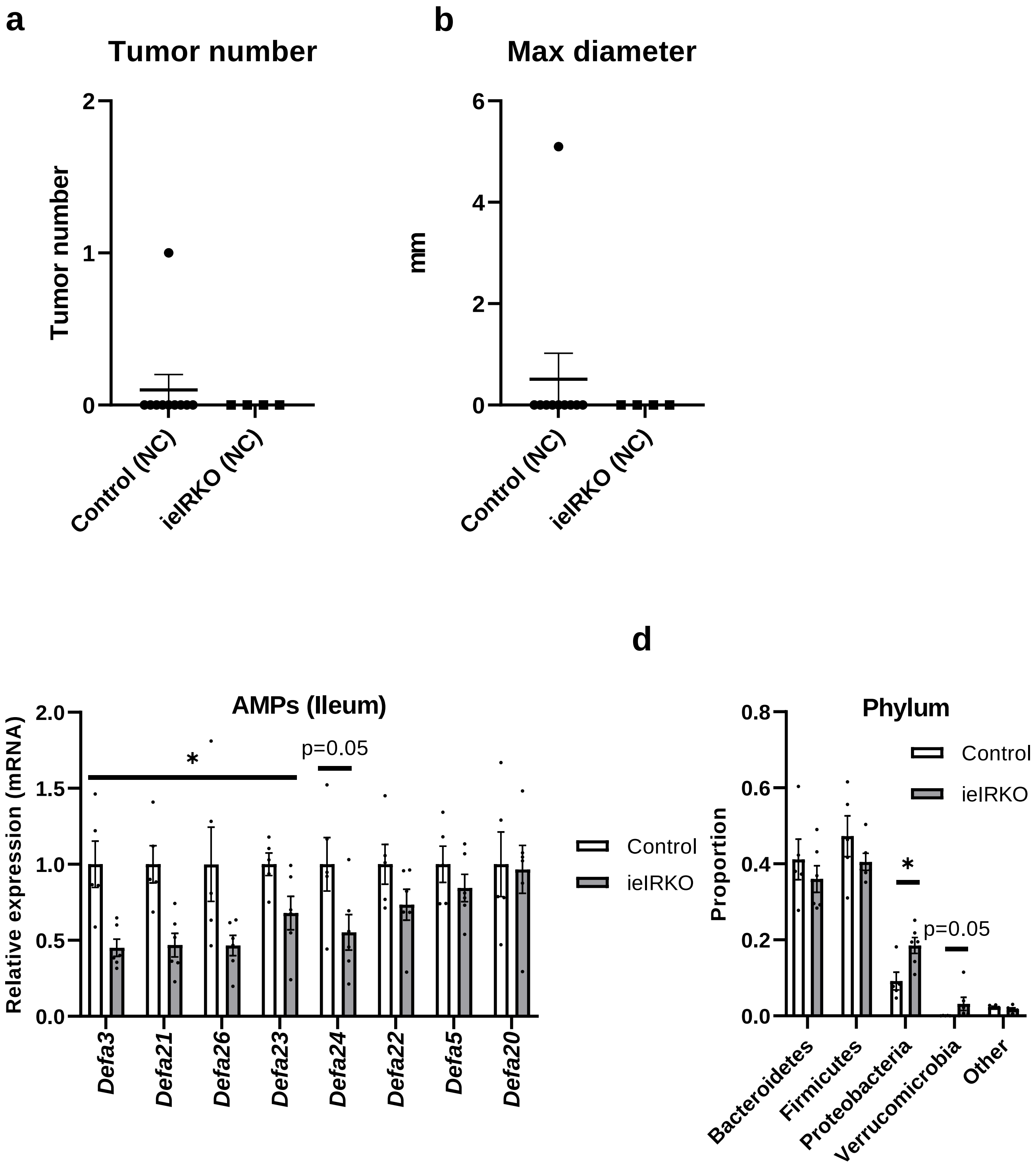

Supplementary Figure 16, Related to Figure 9 Phenotype of 18-month-old ieIRKO-DIO mice of liver, ileum and gut flora.
Tumor number (**a**) and maximum diameter (**b**) in individual intestinal epithelial insulin receptor knock-out (ieIRKO) mice bred with normal chow diet. Control n = 10, ieIRKO n = 4
Relative expression of AMPs of HFD-fed ieIRKO mice at 18 months of age (**c**). *$P < 0.05$, by 2-sided unpaired t test (**c**).
Phylum level analyses determined by 16S metagenomics (**d**). Statistical analysis was performed by Control n = 5, ieIRKO n = 6, *$P < 0.05$, 2-sided unpaired t test (*Proteobacteria*), Mann-Whitney's U test (*Verrucomicrobia*) (**d**). Values of the data are expressed as mean ± SEM (**a, b, c, d**). The exact P values are provided in **Supplementary Data 3**.

Unropped Images Related to Supplementary Figure 3h

Anti-pHSL

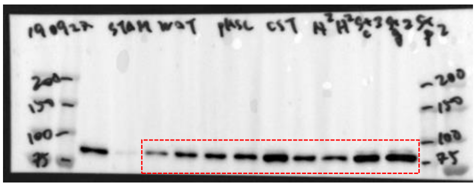

Anti-tHSL

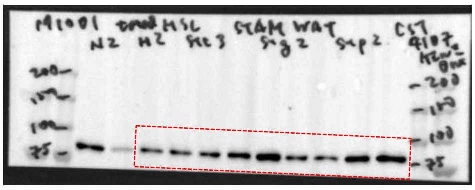

Anti-actin (The membrane was turned over)

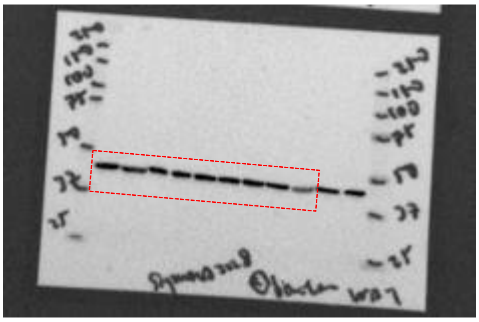

Uncropped Images Related to Supplementary Figure 14a

Anti-pAkt

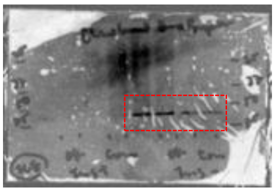

Anti-tAkt

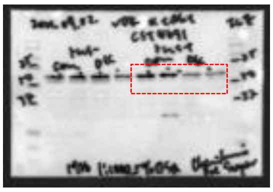

Anti-pS6

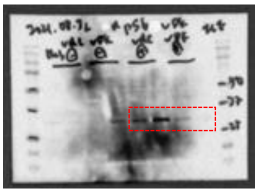

Anti-tS6

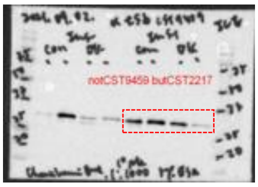

Anti-actin

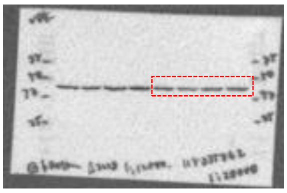

Uncropped Images Related to Supplementary Figure 14c

Anti-pAkt

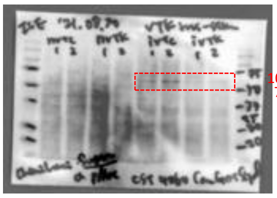

Anti-tAkt

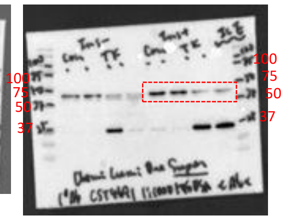

Anti-pS6

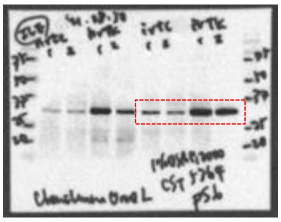

Anti-tS6

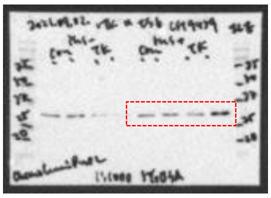

Anti-actin

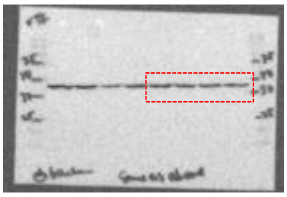

Supplementary Figure 17, Related to Supplementary Figure 3, 14 Uncropped images for western blotting in **Supplementary Figures**.

Supplementary Figure 17

Target	Normal		STAM-NON		STAM-INS	
	Average	S.E.	Average	S.E.	Average	S.E.
FA(16:3)	Undetermined $	N.D. #	2.77044E-05	N.D. #	Undetermined $	N.D. #
Linolenic acid	2.65331E-05	3.5358E-06	0.003885604	0.002847185	0.000959049	0.000172009
Oleoyl ethanolamine	0.000137415	4.46835E-05	0.008018037	0.002716507	0.002260336	0.000418432
Linoleic acid	0.000443608	0.000106296	0.023498003	0.003807799	0.012597761	0.003404501
FA(22:5)	0.000606305	3.67642E-05	0.015770329	0.010453361	0.00263598	0.000484277
Heptadecanoic acid	6.12893E-05	2.60408E-06	0.001038575	0.000202834	0.000432732	6.37901E-05
Palmitic acid	0.000512008	8.13001E-06	0.005939604	0.000850581	0.002668527	0.000320019
FA(12:0)	1.6416E-05	9.13126E-07	0.000104572	6.58382E-05	3.74038E-05	4.01712E-06
AC(12:1)	7.54535E-05	N.D. #	0.000470638	N.D. #	0.00015094	3.5995E-05
Taurochenodeoxycholic acid	0.001040839	0.000148605	0.005860696	0.003484175	0.002710993	0.000938136
Taurolithocholic acid	4.60261E-05	7.82902E-06	0.000218452	0.000185166	5.08851E-05	3.11533E-06
Riboflavin	3.57813E-05	2.83795E-06	0.000130039	5.67362E-05	5.13996E-05	1.68877E-06
Palmitoylethanolamide	0.000447364	0.000145081	0.000896404	0.00036137	0.000464475	5.62052E-05

Supplementary Table 1, Related to Figure 4 Abundance of metabolites determined by LC-MS in liver tissue of normal mice, non-treated STAM mice (STAM-NON), insulin-treated STAM mice (STAM-INS) at 20 weeks of age.
"S.E." means standard error. $; "Undetermined" means below the detection limit. #; "N.D." means the value was not calculated because one or more values of samples were below the detection limit.

	Discovery?	P value	Mean of Normal	Mean of STAM-NON	Difference	SE of difference	t ratio	df	q value
O_sinus	No	0.466375	0.01708	0.01164	0.005445	0.007069	0.7702	7	0.18655
P_distasonis	Yes	0.028559	0.002377	0.01344	-0.01106	0.004024	2.749	7	0.017951
B_hansenii	No	0.862087	0.01431	0.01583	-0.001517	0.008416	0.1802	7	0.291783
B_rodentium	Yes	0.005797	0.001289	0.01722	-0.01594	0.004072	3.914	7	0.005101
A_crotonatoxidans	No	0.351546	0.01404	0.02002	-0.005975	0.005988	0.9979	7	0.15468
R_gnavus	Yes	0.010574	0.007389	0.02422	-0.01683	0.004866	3.458	7	0.007754
B_sartorii	Yes	0.000112	0.004167	0.03875	-0.03458	0.004466	7.744	7	0.000247
P_buccalis	Yes	0.043903	0.004991	0.04197	-0.03698	0.01508	2.453	7	0.021463
B_coccoides	No	0.735221	0.05906	0.04862	0.01044	0.02968	0.352	7	0.269581
P_goldsteinii	Yes	0.000048	0.3438	0.08065	0.2632	0.0298	8.83	7	0.000212
B_acidifaciens	Yes	0.0003	0.03951	0.1153	-0.07577	0.01145	6.616	7	0.00033
B_massiliensis	Yes	0.000236	0.02073	0.2286	-0.2079	0.03023	6.877	7	0.00033
A_muciniphila	Yes	0.032709	0.05799	0.2367	-0.1787	0.06732	2.655	7	0.01799

Supplementary Table 2, Related to Figure 4 The q value to consider false discovery rate produced by multiple comparison.
The FDR q values were shown. To avoid type I error, two-stage step-up (Benjamini, Krieger, and Yekutieli, FDR = 0.10) analysis was added to the data sets in **Fig. 4c**. The criteria to pick up bacteria was explained in figure legend of **Fig. 4c**.

	Discovery?	P value	Mean of Insulin	Mean of Phlorizin	Difference	SE of difference	t ratio	df	q value
A_crotonatoxidans	No	0.232727	-0.7333	0.03382	-0.7672	0.5781	1.327	6	0.3072
O_sinus	No	0.575557	-0.7543	-1.189	0.4347	0.7345	0.5919	6	0.542668
B_sartorii	Yes	0.012428	-1.361	-0.2432	-1.118	0.317	3.526	6	0.041014
R_gnavus	No	0.062587	-1.523	-0.3416	-1.181	0.5176	2.282	6	0.137691
B_massiliensis	Yes	0.00827	-1.642	-0.3966	-1.246	0.3219	3.87	6	0.041014
B_coccoides	No	0.298653	-1.687	-1.447	-0.2409	0.2117	1.138	6	0.328518
J_ignava	No	0.954388	-2.898	-2.859	-0.0391	0.6557	0.05963	6	0.78737
B_hansenii	No	0.183217	-2.998	-1.793	-1.205	0.801	1.504	6	0.302308

Supplementary Table 3, Related to Figure 4 The q value to consider false discovery rate (FDR) produced by multiple comparison.
The FDR q values were shown. To avoid type I error, two-stage step-up (Benjamini, Krieger, and Yekutieli, FDR = 0.10) analysis was conducted to the data sets in **Fig. 4d**. The criteria to pick up bacteria was explained in figure legend of **Fig. 4d**.

	Insulin treatment	Age	BMI [cm/m^2]	AST [IU/L]	ALT[IU/L]	TC [mg/dL]	TG[mg/dL]
Average	No insulin use	73.13333	28.67333	42.06667	38.06667	175.7857	118.7143
	Insulin use	65.6	31.56	61.8	61.6	157.2	95.6
Standard Error	No insulin use	2.922235	1.507277	7.648173	7.159599	10.09785	12.63012
	Insulin use	4.132191	8.164068	36.7141	46.05242	6.318623	21.27675
N	No insulin use	15	15	15	15	14	14
	Insulin use	5	5	5	5	5	5
p value		0.17772	0.549502	0.353287	0.360329	0.191748	0.373174

Pathological Stage (Matteoni 4 or NASH LC); P = 0.6126 (Fisher's exact test)
Sex; P = 0.6126 (Fisher's exact test)

Supplementary Table 4, Related to Figure 5 Comparisons of potential confounding factors in this study.
The statistical significance between the groups (insulin use / no insulin use) assessed by t test (non-categorical parameter) and Fisher's exact test (categorical parameter). The parameters in which the number of missing values were more than three were not selected.

		OGTT_0min [mg/dL]	OGTT_30min [mg/dL]	OGTT_60min [mg/dL]	OGTT_120min [mg/dL]	Cholic acid [µM]	Deoxycholic acid[µM]
Average	No insulin use	102.75	218	285.25	319.5	0.2125	0.4375
	Insulin use	175.5	237	272	302.5	0.433333333	0.933333333
Standard Error	No insulin use	8.267297883	7.538788585	14.78949515	22.68810261	0.163048525	0.221953583
	Insulin use	27.52120395	38	66	128.5	0.384418753	0.643773597
N	No insulin use	8	4	4	4	8	8
	Insulin use	4	2	2	2	3	3

Supplementary Table 5, Related to Figure 5 Aggregate data of blood glucose level in OGTT and bile acid level in this study

Supplementary Table 5

taxon	Fold change (Natural Logarithm)	[-log10 p value]	[-log10 q value]
Bacteroides Bacteroides_uniformis(AB050110)	-1.28065694	0.480456195	0
Bacteroides Bacteroides_fragilis(CR626927)	0.010968259	0.002203529	0
Enterobacter Enterobacter_tabaci(NR_146667.1)	-3.124653512	1.042578193	0
Klebsiella Klebsiella_variicola(AJ783916)	-3.939992378	2.090698161	0
Bacteroides Bacteroides_vulgatus(CP000139)	-2.246559113	0.985761217	0
Bacteroides Bacteroides_dorei(AB242142)	-0.720403034	0.203555751	0
Alistipes Alistipes_onderdonkii(AY974071)	-1.402169225	0.443895356	0
Parabacteroides Parabacteroides_distasonis(AB238922)	-0.524395856	0.148877188	0
Akkermansia Akkermansia_muciniphila(AY271254)	1.323291277	0.36260933	0
Faecalibacterium Faecalibacterium_prausnitzii(AJ413954)	0.778709913	0.907268913	0
Bacteroides Bacteroides_thetaiotaomicron(AE015928)	-1.519860218	0.646641553	0
Bacteroides Bacteroides_ovatus(AB050108)	-0.679220854	0.165796408	0
Bacteroides Bacteroides_caccae(X83951)	-0.603644086	0.202863328	0
Parabacteroides Parabacteroides_merdae(AB238928)	-1.515700133	0.501682925	0
Clostridium_XlVa Lachnoclostridium_pacaense(NR_147396.1)	-0.435752328	0.110220902	0
Alistipes Alistipes_senegalensis(NR_118219.1)	-1.10943633	0.345893397	0
Collinsella Collinsella_aerofaciens(NR_113316.1)	-1.490094846	0.452828241	0
Prevotella Prevotella_copri(AB064923)	-1.210336309	0.376881775	0
Phascolarctobacterium Phascolarctobacterium_faecium(X72865)	-2.181269123	0.835991318	0
Megasphaera Megasphaera_elsdenii(NR_102980.1)	-1.088325808	0.341088135	0

Supplementary Table 6, Related to Figure 5 16S metagenomic signature alteration by insulin use in NASH patients with diabetes

The coordinates of the volcano plot of proportion to total reads of fold change by insulin treatment, p value and q value by ANCOM-BC2 test between the two groups (see also **Fig. S8A**).
