# Supplementary material for: Gut insulin action protects from hepatocarcinogenesis in diabetic mice comorbid with nonalcoholic steatohepatitis
Source: Nat Commun. 2023 Oct 18;14:6584. doi: 10.1038/s41467-023-42334-y (PMC10584811; doi:10.1038/s41467-023-42334-y)
Supplement: Supplementary file 3 — Description of Additional Supplementary Files [file 41467_2023_42334_MOESM3_ESM.pdf]

Description of Additional Supplementary Files

1. Supplementary Data 1: Primer sets used in this study.

2. Supplementary Data 2: STORMS checklist for human 16S metagenomic study.

3. Supplementary Data 3; List of *P* value calculated in this study.
