# Supplementary material for: Gut insulin action protects from hepatocarcinogenesis in diabetic mice comorbid with nonalcoholic steatohepatitis
Source: Nat Commun. 2023 Oct 18;14:6584. doi: 10.1038/s41467-023-42334-y (PMC10584811; doi:10.1038/s41467-023-42334-y)
Supplement: Supplementary file 7 — Reporting Summary [file 41467_2023_42334_MOESM7_ESM.pdf]

nature portfolio

Corresponding author(s): Kotaro Soeda

Last updated by author(s): Aug 17, 2023

Reporting Summary

Nature Portfolio wishes to improve the reproducibility of the work that we publish. This form provides structure for consistency and transparency in reporting. For further information on Nature Portfolio policies, see our Editorial Policies and the Editorial Policy Checklist.

Statistics

For all statistical analyses, confirm that the following items are present in the figure legend, table legend, main text, or Methods section.

n/a	Confirmed	
☐	☒	The exact sample size (*n*) for each experimental group/condition, given as a discrete number and unit of measurement
☐	☒	A statement on whether measurements were taken from distinct samples or whether the same sample was measured repeatedly
☐	☒	The statistical test(s) used AND whether they are one- or two-sided<br>*Only common tests should be described solely by name; describe more complex techniques in the Methods section.*
☒	☐	A description of all covariates tested
☐	☒	A description of any assumptions or corrections, such as tests of normality and adjustment for multiple comparisons
☐	☒	A full description of the statistical parameters including central tendency (e.g. means) or other basic estimates (e.g. regression coefficient) AND variation (e.g. standard deviation) or associated estimates of uncertainty (e.g. confidence intervals)
☐	☒	For null hypothesis testing, the test statistic (e.g. *F*, *t*, *r*) with confidence intervals, effect sizes, degrees of freedom and *P* value noted<br>*Give P values as exact values whenever suitable.*
☒	☐	For Bayesian analysis, information on the choice of priors and Markov chain Monte Carlo settings
☒	☐	For hierarchical and complex designs, identification of the appropriate level for tests and full reporting of outcomes
☒	☐	Estimates of effect sizes (e.g. Cohen's *d*, Pearson's *r*), indicating how they were calculated

Our web collection on statistics for biologists contains articles on many of the points above.

Software and code

Policy information about availability of computer code

Data collection	Illumina HiSeq 2500 (Whole exome sequencing), Illumina MiSeq (16S metagenomics), Illumina NovaSeq (RNA sequencing), Agilent CE-TOFMS system (Agilent Technologies), Agilent LC-TOF/MS system (Agilent Technologies)
Data analysis	To draw graphs and to analyse we used GraphPad Prism (version 9.5.0). BaseSpace 16S Metagenomics App (version 1.1.0 , Illumina), EZ-R (version 4.2.2), ANCOM-BC2 (ver2.0.2), TRRUST (version 2), karkinos (version 4.1.11), DAVID (version 6.8), MasterHands (version 2.16.0.15, version 2.17.1.11, HMT, developed by Keio University), SampleStat (version 3.14, HMT), ImageJ (version 1.52), CLC genomics workbench (version 21.0.3), was used in this study.

For manuscripts utilizing custom algorithms or software that are central to the research but not yet described in published literature, software must be made available to editors and reviewers. We strongly encourage code deposition in a community repository (e.g. GitHub). See the Nature Portfolio guidelines for submitting code & software for further information.

Data

Policy information about availability of data

All manuscripts must include a data availability statement. This statement should provide the following information, where applicable:

- Accession codes, unique identifiers, or web links for publicly available datasets
- A description of any restrictions on data availability
- For clinical datasets or third party data, please ensure that the statement adheres to our policy

The whole exome sequencing and metagenomic data from mice generated in this study have been deposited in the SRA database under accession code PRJNA866345 [https://dataview.ncbi.nlm.nih.gov/object/PRJNA866345?reviewer=egt5015oc8n6ir16q44ton2mdv]. The RNA sequencing data from the ileum of mice generated in this study have been deposited in the GEO database under accession code GSE210876 [https://www.ncbi.nlm.nih.gov/geo/query/acc.cgi?acc=GSE210876]. The microarray data from the liver of mice generated in this study have been deposited in the GEO database under accession code GSE210517 [https://www.ncbi.nlm.nih.gov/geo/query/acc.cgi?acc=GSE210517]. The human metagenomic data generated in this study have been deposited in the DDBJ database under study accession code JGAS000574 and dataset code JGAD000700 [https://humandbs.biosciencedbc.jp/en/hum0371-v1]. To obtain data set in DDBJ, data users need to apply an application for Using NBDC Human Data to reach the Controlled-access Data, following the "Ethical Guidelines for Life Science and Medical Research Involving Human Subjects," which is based on Japan's Personal Information Protection Law. How to access is shown in the following URL; https://humandbs.biosciencedbc.jp/en/data-use. Source data are provided in this paper.
Mouse genome MGSCv37/mm9 (for Whole exome sequencing), GRCm38/mm10 (for RNA sequencing), Greengenes (for 16S metagenomics) were used for reference.reasonable request. The results of RNA sequencing, DNA microarray, whole exome sequencing and 16S metagenomics of mice (SRA; PRJNA866345, GEO; GSE210517, GEO; GSE210876) and 16S metagenomics in human study (DDBJ; Study: JGAS000574, Dataset: JGAD000700.) are deposited.

Human research participants

Policy information about studies involving human research participants and Sex and Gender in Research.

Reporting on sex and gender	Self-reported sex data were indicated in supplementary table.
Population characteristics	Age and other profiles of participants were indicated in supplementary table 4 and 5.
Recruitment	16S metagenomic analysis was performed on stool DNA samples collected from 27 patients who experienced liver biopsy according to the following criteria or clinically diagnosed severe non-B non-C cirrhosis at the University of Tokyo Hospital from 2012 to 2019. The recruitment criteria for liver biopsy in patients without HCC were previously described by an associate researcher. (ref39: Sci rep 9, 10663, doi:10.1038/s41598-019-47216-2)
Ethics oversight	The University of Tokyo Medical Research Center Ethics Committee

Note that full information on the approval of the study protocol must also be provided in the manuscript.

Field-specific reporting

Please select the one below that is the best fit for your research. If you are not sure, read the appropriate sections before making your selection.

☒ Life sciences ☐ Behavioural & social sciences ☐ Ecological, evolutionary & environmental sciences

For a reference copy of the document with all sections, see nature.com/documents/nr-reporting-summary-flat.pdf

Life sciences study design

All studies must disclose on these points even when the disclosure is negative.

Sample size	Early study of STAM mice showed the data in which the expression profile of liver in n = 3 (Medical Molecular Morphology volume 46, pages141–152 (2013)), comparing STAM mice with normal mice. In the previous study using glucose-lowering agent, improvements in the NASH phenotype was observed in n = 7 after 4-week treatment (Medical Molecular Morphology volume 47, pages137–149 (2014)). From the results in these previous studies, we planed normal vs. STAM study in n =4-6, 3-week intervention study in n = 4-6, and long-term (14 weeks) study to observe in tumor-evident phase in n =10-14, respectively.
Data exclusions	To generate carcinogenesis models, mice in which administration was technically failed were excluded, whereas all data were included in the other experiments.
Replication	Experiments in this study were supported by one additional experiment, whereas experiments requiring a duration of follow-up for over one year and the human study were performed in a single cohort. All attempts at replication were successful.
Randomization	Mice were assigned to intervention by the data of body weight and blood glucose. In ex vivo experiments, one dissected ileum was from a mouse for biological replicates. Mice were assigned to intervention by the data of body weight and blood glucose. In ex vivo experiments, one dissected ileum was taken from one mouse to obtain biological replicates.

Blinding

The investigators were blinded to the genotype of mice or intervention to which mice were subjected when assessing macroscopic and histological analyses of hepatocarcinogenesis. In ex vivo experiments, the investigators could not be blinded to the group allocation because the investigators must know the treatment for each group to select appropriate medium. For western blot, qPCR analyses, and next generation sequencing, the investigators were not blinded because these experiments were conducted by the same person as one who collected the samples.

Reporting for specific materials, systems and methods

We require information from authors about some types of materials, experimental systems and methods used in many studies. Here, indicate whether each material, system or method listed is relevant to your study. If you are not sure if a list item applies to your research, read the appropriate section before selecting a response.

Materials & experimental systems

n/a	Involved in the study	
☐	☒	Antibodies
☒	☐	Eukaryotic cell lines
☒	☐	Palaeontology and archaeology
☐	☒	Animals and other organisms
☒	☐	Clinical data
☒	☐	Dual use research of concern

Methods

n/a	Involved in the study	
☒	☐	ChIP-seq
☒	☐	Flow cytometry
☒	☐	MRI-based neuroimaging

Antibodies

Antibodies used

Primary antibodies: Hif1a (1:1000, Novus, #NB100-105), pHSL (1:1000, Cell Signaling Technology, #4139), tHSL (1:1000, Cell Signaling Technology, CST#4107), pS6 (1:2000, Cell Signaling Technology, #5364), tS6 (1:1000, Cell Signaling Technology, #2217), pAkt (1:2000, Cell Signaling Technology, #4060), and tAkt (1:1000, Cell Signaling Technology, #4691), beta actin (1:12500, Sigma, #A4448)beta actin (Sigma #A2228, 1:12500).
Secondary antibodies: mouse anti-rabbit IgG-HRP: (SantaCruz #sc-2357, 1:5000), Peroxidase AffiniPure Goat Anti-Mouse IgG (H+L) (Jackson, 115-035-062, 1:20000).

Validation

All the immunoblotting experiments were performed on mouse tissues in this study. All antibodies used in this study are commercially available and are stated to be tested by the manufacturer for species reactivity to mouse. The statements and validation data for each primary and secondary antibody for the species and application are also available on the manufacturer's website.
pAkt (Cell Signaling Technology #4060)
https://www.cellsignal.jp/products/primary-antibodies/phospho-akt-ser473-d9e-xp-rabbit-mab/4060
tAkt (Cell Signaling Technology #4691)
https://www.cellsignal.jp/products/primary-antibodies/akt-pan-c67e7-rabbit-mab/4691
Hif1a (Novus #NB100-105)
https://www.novusbio.com/products/hif-1-alpha-antibody-h1alpha67_nb100-105
pHSL (Cell Signaling Technology #4139)
https://www.cellsignal.jp/products/primary-antibodies/phospho-hsl-ser563-antibody/4139
tHSL (Cell Signaling Technology #4107)
https://www.cellsignal.jp/products/primary-antibodies/hsl-antibody/4107
pS6 (Cell Signaling Technology #5364)
https://www.cellsignal.jp/products/primary-antibodies/phospho-s6-ribosomal-protein-ser240-244-d68f8-xp-rabbit-mab/5364
tS6 (CST#2217)
https://www.cellsignal.jp/products/primary-antibodies/s6-ribosomal-protein-5g10-rabbit-mab/2217
Monoclonal Anti-b-Actin (Sigma #A2228)
https://www.sigmaaldrich.com/JP/ja/product/sigma/a2228
Mouse anti-rabbit IgG-HRP (Santa Cruz #sc-2357)
https://www.scbt.com/ja/p/mouse-anti-rabbit-igg-hrp
Peroxidase AffiniPure Goat Anti-Mouse IgG (H+L)
https://www.jacksonimmuno.com/catalog/products/115-035-062

Animals and other research organisms

Policy information about studies involving animals; ARRIVE guidelines recommended for reporting animal research, and Sex and Gender in Research

Laboratory animals

Animal experimental procedures were approved by the Institutional Animal Care and Use Committee of the National Center for Global Health and Medicine (approved protocol number; Med-P16-113, Med-P16-114) and the Animal Care Committee of the University of Tokyo (approved protocol number; 21077, 2022-A044).
Mice (C57BL/6J) were used in this study, from 6 weeks to 18 months of age. C57BL/6J mice were purchased from CLEA Japan. Villin-Cre mice were purchased from Jackson laboratory. They were crossed with insulin receptor (IR)-floxed mice, Akt1-floxed mice, Akt2-floxed mice, TSC2-floxed mic to obtain intestinal epithelium specific gene knock out mice. These floxed mice were provided by

coauthor (Toda G, Mol Cell. 2020 Jul 2;79(1):43-53.e4.). Villin-Cre; IR-floxed was used from their birth to 20 weeks of age. Villin-Cre; Akt1/2-double floxed and Villin-Cre; Akt1/2TSC2-triple floxed mice were used at the age of 8 weeks.
All mice were housed under a 12-hour light/12-hour dark cycle at macroenvironmental temperature and humidity ranges of 20 to 22 °C and 40 % to 60% respectively, and had free access to sterile water and pellet food, unless otherwise indicated.

Wild animals	The study did not involve wild animals.
Reporting on sex	All the mice used in this study were male.
Field-collected samples	The study did not involve samples collected from the field.
Ethics oversight	Animal experimental procedures were approved by the Institutional Animal Care and Use Committee of the National Center for Global Health and Medicine and the Animal Care Committee of the University of Tokyo.

Note that full information on the approval of the study protocol must also be provided in the manuscript.
